# Salt inducible kinases 2 and 3 are required for thymic T cell development

**DOI:** 10.1038/s41598-021-00986-0

**Published:** 2021-11-03

**Authors:** Meriam Nefla, Nicola J. Darling, Manuel van Gijsel Bonnello, Philip Cohen, J. Simon C. Arthur

**Affiliations:** 1 Division of Cell Signalling and Immunology, Wellcome Trust Building, School of Life Sciences, University of Dundee, Dundee, DD1 5EH UK; 2 MRC Protein Phosphorylation and Ubiquitylation Unit, Sir James Black Centre, School of Life Sciences, University of Dundee, Dundee, DD1 5EH UK

**Keywords:** Adaptive immunity, Cellular immunity, Immunology, T cells, Cell signalling, Kinases

## Abstract

Salt Inducible Kinases (SIKs), of which there are 3 isoforms, are established to play roles in innate immunity, metabolic control and neuronal function, but their role in adaptive immunity is unknown. To address this gap, we used a combination of SIK knockout and kinase-inactive knock-in mice. The combined loss of SIK1 and SIK2 activity did not block T cell development. Conditional knockout of SIK3 in haemopoietic cells, driven by a Vav-iCre transgene, resulted in a moderate reduction in the numbers of peripheral T cells, but normal B cell numbers. Constitutive knockout of SIK2 combined with conditional knockout of SIK3 in the haemopoietic cells resulted in a severe reduction in peripheral T cells without reducing B cell number. A similar effect was seen when SIK3 deletion was driven via CD4-Cre transgene to delete at the DP stage of T cell development. Analysis of the SIK2/3 Vav-iCre mice showed that thymocyte number was greatly reduced, but development was not blocked completely as indicated by the presence of low numbers CD4 and CD8 single positive cells. SIK2 and SIK3 were not required for rearrangement of the TCRβ locus, or for low level cell surface expression of the TCR complex on the surface of CD4/CD8 double positive thymocytes. In the absence of both SIK2 and SIK3, progression to mature single positive cells was greatly reduced, suggesting a defect in negative and/or positive selection in the thymus. In agreement with an effect on negative selection, increased apoptosis was seen in thymic TCRbeta high/CD5 positive cells from SIK2/3 knockout mice. Together, these results show an important role for SIK2 and SIK3 in thymic T cell development.

## Introduction

T cell development is a tightly regulated process that requires the precise temporal control of genes needed for the differentiation of T cells^1–3^. Salt Inducible Kinases (SIK) are a group of three protein kinases that have been linked to the transcriptional regulation of a number of genes involved in multiple processes including metabolism and innate immunity^4–6^, but their role in T cell development has not been addressed. SIK1 was first identified in the adrenocortical tissues of rats fed on a high salt diet^7^ and, together with the closely related SIK2 and SIK3 isoforms, they form a sub-group of the AMPK-related family of protein kinases (reviewed in^8^). SIKs are widely expressed, SIK2 and SIK3 being responsible for the great majority of SIK activity in most cell types^9^.

In order to be active, SIKs require phosphorylation of their activation loop by the protein kinase LKB1. This appears to occur constitutively in most cells, suggesting that SIKs are not activated in vivo via increased phosphorylation at this site^10^. Consistent with this notion, stimuli have not been identified that increase the intrinsic catalytic activity of SIKs, suggesting that regulation may occur at the level of SIK protein expression or via stimuli that inhibit SIKs. For example, phosphorylation of SIKs by cyclic AMP-dependent protein kinase (PKA), correlates with decreased SIK activity in cells, as judged by the dephosphorylation of SIK substrates^11,12^. How PKA-mediated phosphorylation decreases SIK activity is not yet fully understood, but it is possible that it may promote interaction with 14-3-3 proteins and thereby prevent SIKs from recognising their substrates in cells^12,13^.

The first substrates of the SIKs to be identified were members of the cAMP-response element binding protein (CREB)-regulated transcriptional co-activator (CRTC) family^14^. CRTCs bind to the Basic Leucine Zipper (bZIP) domain of CREB, thereby promoting the transcription of CREB-dependent immediate early genes^15,16^. The SIK-catalysed phosphorylation of CRTCs also induces their interaction with 14–3-3 proteins and nuclear exit, while dephosphorylation of CRTCs results in their accumulation in the nucleus^14,17,18^. For these reasons the suppression of SIK activity with small molecule inhibitors induces CRTC dephosphorylation and translocation to the nucleus where it binds to CREB and promotes the transcription of CREB-dependent genes^9,17^. SIKs have also been shown to phosphorylate class IIa Histone Deacetylases (HDACs), promoting 14-3-3 binding and cytoplasmic localisation^19,20^.

In the immune system, the role of SIKs has mainly been studied in the context of innate immune cells and Toll-Like Receptor (TLR) signalling, where SIK inhibition limits the production of inflammatory mediators^11,17,21–23^. In macrophages, production of the anti-inflammatory cytokine Interleukin (IL)-10 in response to TLR agonists is dependent on the phosphorylation of CREB by Mitogen and Stress-activated Protein Kinase 1 (MSK1) and MSK2^24–26^. TLRs do not directly modify SIK catalytic activity or induce CRTC3 re-localisation to the nucleus. However, co-stimulation with TLR agonists and secondary stimuli that activate the cAMP–PKA pathway, such as Prostaglandin E_2_ (PGE_2_), blocks SIK-mediated CRTC3 phosphorylation^11^, promoting CRTC3 dephosphorylation and translocation to the nucleus where it acts as a co-activator for CREB on the IL-10 promoter. In addition, SIK inhibition promotes the transcription of several other genes linked to potential anti-inflammatory phenotypes in macrophages, including Arginase 1 and Sphingosine Kinase 1^11,17^.

The potential roles of SIKs in adaptive immunity have not been addressed, although the SIK substrate CRTC2 has been reported to be involved in regulating T-helper cell 17 (Th17) development^27^. This research showed that CRTC2 was not required for T cell development in the thymus, but the ability of CD4 T cells to produce IL-17 under Th17 polarising conditions was reduced in the absence of CRTC2. In line with this, Th17 numbers were reduced in CRTC2 knockout mice relative to wild type animals in experimental autoimmune encephalitis and the CRTC2 knockout mice were protected in this model^27^. In addition to IL-17, CREB has also been linked to the transcription of a number of other genes important in T cells including CD4^28^, CD8^29^ and enhancers of T cell receptor (TCR) genes^30,31^, while CREB is known to be phosphorylated downstream of TCR signaling by MSK1/2^32^.

In this paper we have studied how the loss of each SIK isoform or its catalytic activity affects T cell development. We report that SIK1 and SIK2 activity are dispensable for normal T cell development, whereas knockout of SIK3 results in decreased T cell numbers. The combined knockout of SIK2 and SIK3 results in a severe block in thymic T cell development, consistent with a defect in the positive or negative selection of double positive thymocytes.

## Results

### Loss of SIK3 reduces peripheral T cell number in vivo

**Figure 1:**
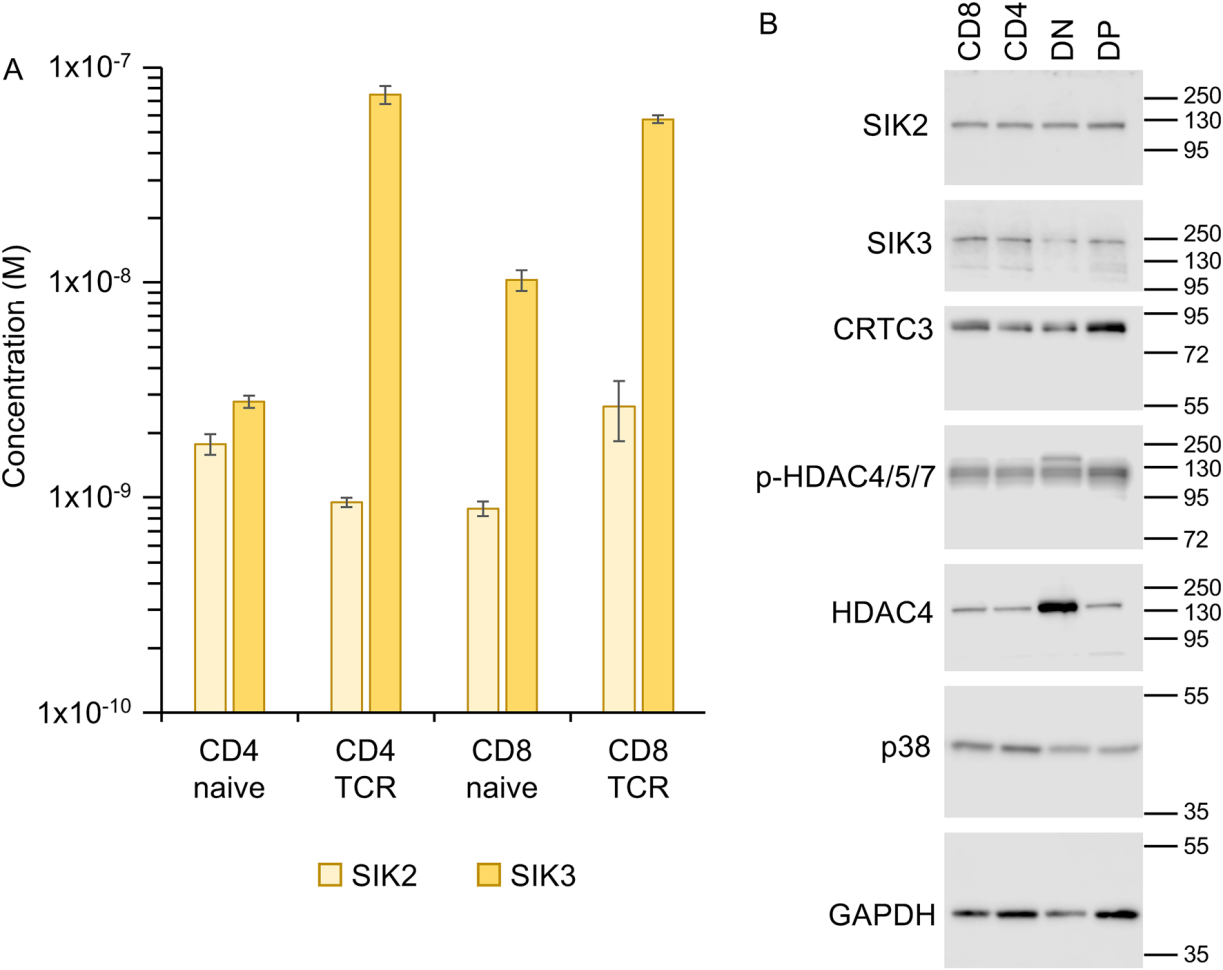
SIK levels in T cell subsets. (**A**) Naïve CD4 and CD8 T cells were isolated by FACS and subject to TMT labelled proteomic analysis as described in^33^. For ex vivo stimulations of the TCR, GP33 (glycoprotein amino acids 33–41), 20 ng/ml IL-2 and 2 ng/ml IL-12 were used to stimulate CD8 cells from P14 mice. OVA peptide loaded onto antigen presenting cells was used to stimulate CD4 cells from OT-II mice as described in^33^. Graphs show mean and standard deviation for SIK2 and SIK3. SIK1 was not detected. For CD4 cells, data are from independent preparations from 3 mice per genotype and for CD8 cells from 6 mice per genotype. Data is derived from Howden et al. 2019, Nat. Immunol. 20, 1542–1554^33^. (**B**) Thy1^+ve^ thymocytes pooled from 5 mice were sorted based on CD4 and CD8 expression into CD8 single positive, CD4 single positive, double negative (DN) and CD4/8 double positive (DP) cells by FACS. 5 μg of lysate was separated by SDS-PAGE and blotted using the indicated antibodies. Molecular weight markers are shown, with weights in kDa.

**Figure 2:**
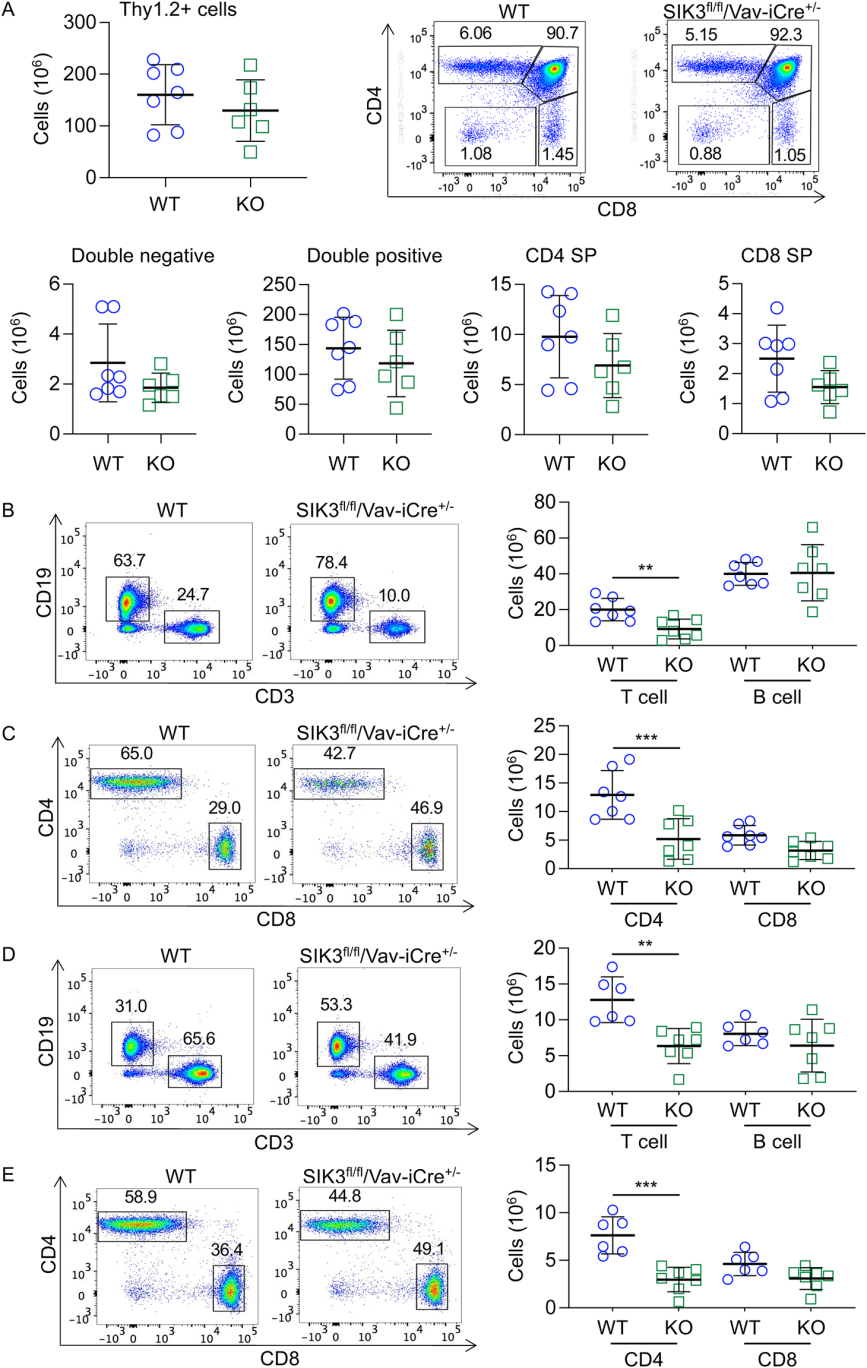
SIK3^fl/fl^/Vav-iCre^+/-^ mice have reduced numbers of T cells in the spleen and lymph nodes. Thymi, spleen and lymph nodes were isolated from wild type (WT) and SIK3^fl/fl^/Vav-iCre^+/-^ (KO) mice and analysed by flow cytometry. (**A**) Representative CD4 / CD8 plots of Thy1.2^+ve^ cells in the thymus and graphs for absolute numbers of Thy1.2^+ve^, DN, DP, CD4 SP and CD8 SP cells. (**B**) Numbers of CD3^+ve^ T cells and CD19^+ve^ B cells in the spleen, along with representative FACS plots. (**C**) Absolute numbers of CD4 and CD8 T cells and representative CD4 / CD8 plots of CD3^+ve^ cells in the spleen. (**D**) Numbers of CD3^+ve^ T cells and CD19^+ve^ B cells in the lymph nodes, along with representative FACS plots. (**E**) Absolute numbers of CD4 and CD8 T cells and representative CD4 / CD8 plots of CD3^+ve^ T cells in the lymph nodes. Graphs show mean with symbols representing measurements from individual mice. Differences between wild type and knockout mice were analysed by Student’s t-test for the Thy1^+ve^ cell graph and by RM two way ANOVA with Sidak’s post hoc testing for the other graphs. *p* < 0.05 is indicated by *, < 0.01 by ** and < 0.001 by ***. Graphs show n = 6 for analysis of thymi, n = 7 or n = 6 for wild type and SIK3 knockouts for spleens and n = 6 for wild type and n = 7 for SIK3 knockout lymph nodes.

Previously, we have shown that SIK activity is high in the thymus and is predominately due to SIK2 and SIK3 with only a minor input from SIK1^9^. Consistent with this finding, proteomic studies on peripheral T cells have established that SIK2 and SIK3 are expressed in T cells, but have failed to detected SIK1^33^. Interestingly, in these experiments SIK3 was strongly upregulated following T cell receptor (TCR) stimulation, suggesting an important role(s) for SIK3 in T cells (Fig. 1A)^33^. Developing T cells in the thymus undergo a series of stages, starting with Double Negative (DN) cells^34,35^. These cells then upregulate CD4 and CD8 to become Double Positive (DP) cells, at which point they start to express the TCR on their surface before losing expression of either CD4 or CD8 to become Single Positive (SP) cells. Analysis of gene expression data in the Immgen database^36^ showed that SIK3 mRNA levels increased at the DP stage, coinciding with the cell surface expression of the TCR (supplementary Fig. 1). A similar pattern was also observed in human T cells in single cell RNAseq data in the Human Cell Atlas (supplementary Fig. 1,^37^). In agreement with this, SIK3 levels were slightly higher in DP cells than DN cells when examined by immunoblotting of sorted thymic subsets (Fig. 1B). We therefore initially assessed the impact of SIK3 knockout on T cell development. Both SIK3 knockout and kinase-inactive knock-in mice displayed increased postnatal mortality, decreased size at weaning and failure to thrive^9,38^. To avoid these adverse phenotypes, a conditional knockout of SIK3 was generated using a Vav-iCre transgene to delete SIK3 selectively in the hematopoietic lineage (SIK3^fl/fl^/Vav-iCre^+/−^ mice, supplementary Fig. 2A). These mice were viable and did not exhibit the overt adverse developmental and welfare issues associated with the total SIK3 kinase-inactive knock-in or knockout mice (data not shown). Thymi from SIK3^fl/fl^/Vav-iCre^+/−^ mice had similar numbers of Thy1.2^+ve^ T cells compared to wild type animals (Fig. 2A). A more detailed analysis of DN, DP and SP cells in the thymi of these mice showed that there were no significant differences in the numbers of these subsets between the wild type and SIK3^fl/fl^/Vav-iCre^+/−^ mice (*p* > 0.05, two way ANOVA and Sidak’s post hoc testing), although there was a trend to a decrease in the numbers of CD4 and CD8 SP cells in the SIK3^fl/fl^/Vav-iCre^+/−^ thymi (Fig. 2A). During development in the thymus DN cells can upregulate CD8 slightly before CD4 to give rise to an Intermediate Single Positive (ISP) population. To ensure that the presence of ISPs was not obscuring a change in CD8 SP cells in the SIK3 knockout, ISPs were excluded by gating on cells with high cell surface TCRβ expression. Analysis of the numbers of TCR^high^ CD4 and CD8 SP cells again showed no significant difference between the wild type and SIK3^fl/fl^/Vav-iCre^+/−^ mice, although the trend for lower numbers of SP cells in the knockout was maintained (supplementary Fig. 3). There was however a reduction in the number of CD3^+ve^ T cells in the spleens of SIK3^fl/fl^/Vav-iCre^+/−^ mice relative to wild type controls (Fig. 2B). In contrast B cell numbers were unaffected by loss of SIK3. The decrease in T cells corresponded to a decrease in both the CD4 and CD8 T cell subsets, although this was more pronounced for the CD4 cells (Fig. 2C). Similar to the spleen, B cell numbers in the lymph nodes did not differ significantly between wild type and SIK3^fl/fl^/Vav-iCre^+/-^ mice, but T cell numbers were decreased (Fig. 2D). Again, this corresponded to a decrease in both CD4 and CD8 T cells (Fig. 2E). Together, these findings suggest either a reduced output of T cells from the thymus in SIK3^fl/fl^/Vav-iCre^+/−^ mice or a decreased survival or entry into the secondary lymphoid organs.

### The catalytic activity of SIK1 and SIK2 are dispensable for T cell development

**Figure 3:**
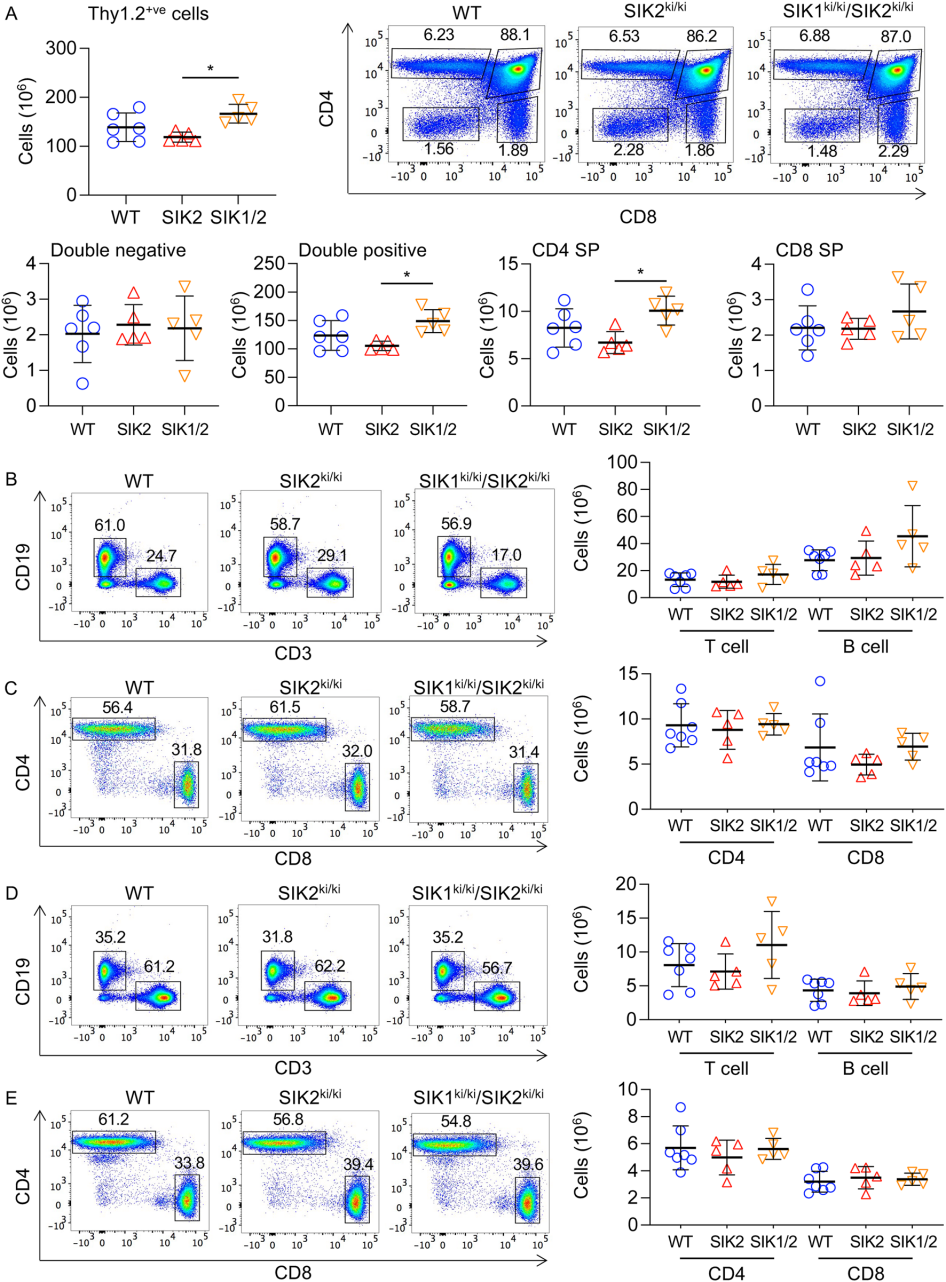
T cell development is not abrogated in SIK2^ki/ki^ and SIK1^ki/ki^ /SIK2^ki/ki^ mice. Thymi (**A**), spleen (**B**, **C**) and lymph nodes (**D**, **E**) were isolated from wild type (WT), SIK2^ki/ki^ (SIK2) and SIK1^ki/ki^/SIK2^ki/ki^ (SIK1/2) mice and analysed by flow cytometry as described in the methods. (**A**) Representative CD4 / CD8 plots of Thy1.2^+ve^ cells in the thymus and graphs for absolute numbers of Thy1.2^+ve^, DN, DP, CD4 SP and CD8 SP cells. (**B**) Numbers of CD3^+ve^ T cells and CD19^+ve^ B cells in the spleen, along with representative FACS plots. (C) Absolute numbers of CD4 and CD8 T cells and representative CD4 / CD8 plots of CD3^+ve^ T cells in the spleen. (**D**) Numbers of CD3^+ve^ T cells and CD19^+ve^ B cells in the lymph nodes, along with representative FACS plots. (**E**) Absolute numbers of CD4 and CD8 T cells and representative CD4 / CD8 plots of CD3^+ve^ cells in the lymph nodes. Graphs show mean with symbols representing measurements from individual mice. Differences in cell number between the different genotypes was analysed by RM two way ANOVA with Sidak’s post hoc testing except for the number of Thy1^+ve^ cells in A which was analysed by one way ANOVA. *p* < 0.05 is indicated by *, < 0.01 by ** and < 0.001 by ***. 5 SIK2^ki/ki^ and SIK1^ki/ki^ /SIK2^ki/ki^ mice were analysed along with 6 wild type thymi and 7 wild type spleens.

Given the above findings indicating a role for SIK3 in T cells, we next examined if the related isoforms, SIK1 and SIK2, might also regulate T cell development. To examine this, T cells from SIK2 (SIK2^ki/ki^) and SIK1/2 (SIK1^ki/ki^/SIK2^ki/ki^) kinase-inactive knock-in mice were analysed. In these mice, the threonine residue in the activation loop of SIK1 or SIK2 that is essential for catalytic activity in vitro and in vivo was mutated to alanine^9,10^. Analysis of the thymi from these mice showed that both SIK2^ki/ki^ and SIK1^ki/ki^/SIK2^ki/ki^ knock-in mice had similar numbers of T cells in the thymus compared to wild type controls (Fig. 3A). Analysis of CD4 and CD8 expression in Thy1.2^+ve^ thymocytes by flow cytometry showed that the ratios and numbers of DN, DP and SP T cells in the thymus were not decreased in the SIK2^ki/ki^ or SIK1^ki/ki^/SIK2^ki/ki^ mice relative to the wild type controls (Fig. 3A). Analysis of the spleens from these mice also showed similar numbers of T and B cells in wild type, SIK2^ki/ki^ and SIK1^ki/ki^/SIK2^ki/ki^ mice (Fig. 3B), as judged by the expression of CD19 and CD3 to identify B cells and T cells respectively. The ratio and numbers of CD4 and CD8 cells in the T cell compartment was also unaffected (Fig. 3C). Similar results were obtained in the lymph nodes (Fig. 3D and E).

### Deletion of both SIK2 and SIK3 blocks T cell development

**Figure 4:**
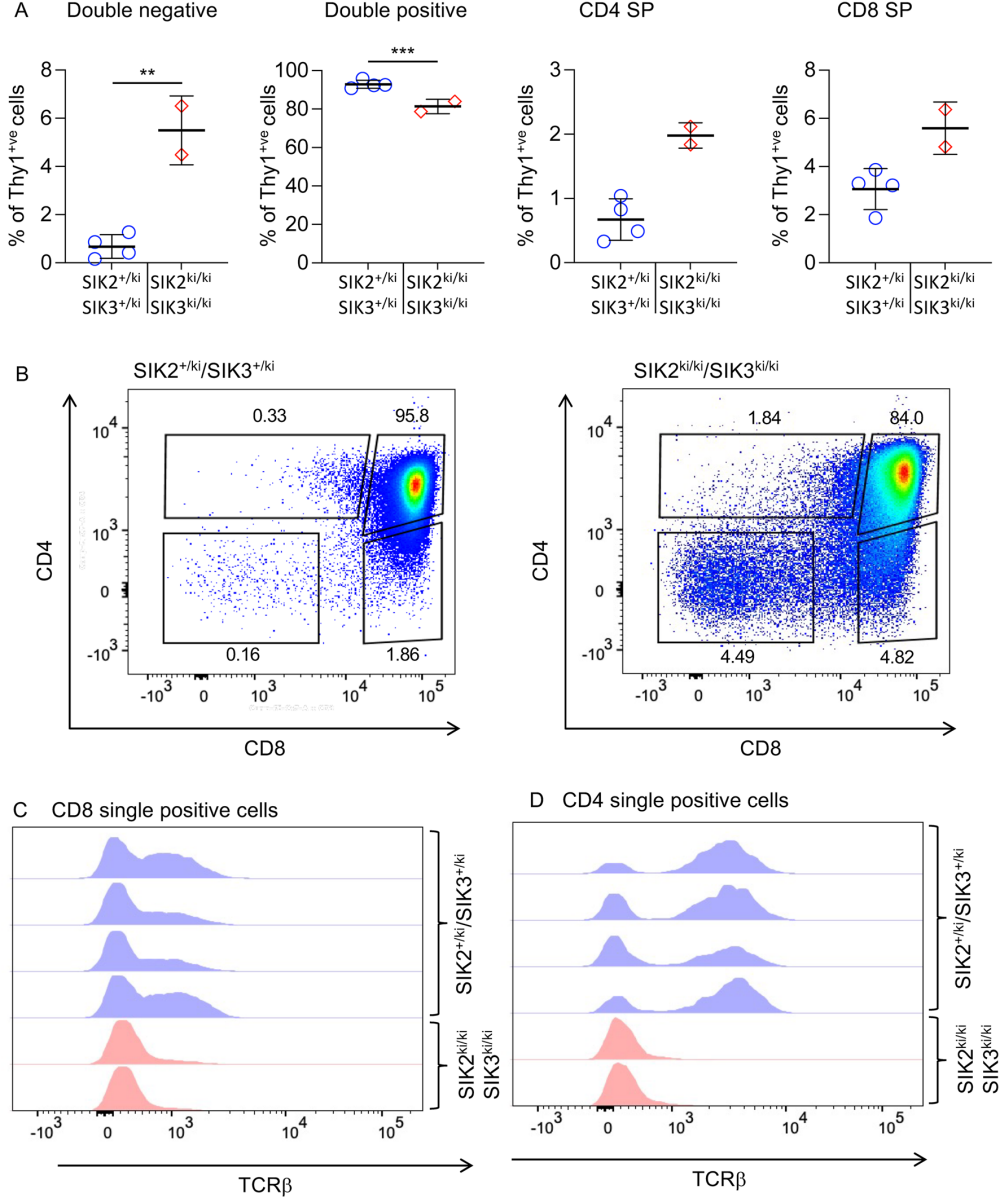
Embryonic T cells in SIK2^ki/ki^/SIK3^ki/ki^ mice have a defect in cell surface TCRβ upregulation. Thymi were isolated from embryonic day 17.5 embryos and analysed by flow cytometry for Thy1, CD4, CD8 and TCRβ. Graphs show percentage of DN, DP and SP cells in in Thy1.2^+ve^ thymocytes from 2 SIK2^ki/ki^/SIK3^ki/ki^ and 4 SIK2^+/ki^/SIK3^+/ki^ littermate controls (**A**) and representative plots are shown in (**B**). Cell surface staining of TCRβ in CD8 single positive (**C**) and CD4 single positive (**D**) thymocytes are also shown. Differences in cell number between the different genotypes was analysed by RM two way ANOVA with Sidak’s post hoc testing. *p* < 0.01 is indicated by ** and < 0.001 by ***.

While loss of SIK2 did not impact T cell development, as T cells express both SIK2 and SIK3 it is possible that compensation can occur between these two isoforms. The SIK2/3 double kinase-inactive knock-in results in late embryonic lethality, with the expected Mendelian frequency of SIK2^ki/ki^/SIK3^ki/ki^ observed at E15.5^9^. To look at the effect on T cell development, embryos were isolated at E17.5 and their thymi excised and analysed by flow cytometry. This showed that SIK2^ki/ki^/SIK3^ki/ki^ thymi had an increased percentage of DN cells and a decreased percentage of DP cells compared to SIK2^+/ki^/SIK3^+/ki^ thymi (Fig. 4A). There was also an increase in the percentage of cells in the CD8 SP gate. Analysis of these cells showed that in the SIK2^ki/ki^/SIK3^ki/ki^ thymi, the majority of these cells were TCRβ low, indicating that they may represent ISP (immature single positive) cells, an intermediate between the DN and DP cells, rather than true CD8 SP cells (Fig. 4B–C). The percentage of CD4 SP cells was also increased, however again none of these cells showed high expression of cell surface TCRβ, suggesting they are not true SP cells that have successfully undergone selection (Fig. 4B, D). Together, this suggests that SIK2 and SIK3 activity is required for the normal progression of T cells through thymic development.

To examine the effect of the loss of SIK2 and SIK3 in the adult thymus, the conditional SIK3 knockout mice were bred to total SIK2 knockout mice and then onto mice carrying a Vav-iCre transgene. The resulting SIK2^-/-^/SIK3^fl/fl^/Vav-iCre^+/-^ mice lack SIK2 in all cells and SIK3 only in hematopoietic cells. Immunoblotting confirmed the absence of both SIK2 and SIK3 in splenocytes from these mice (supplementary Fig. 2B).

**Figure 5:**
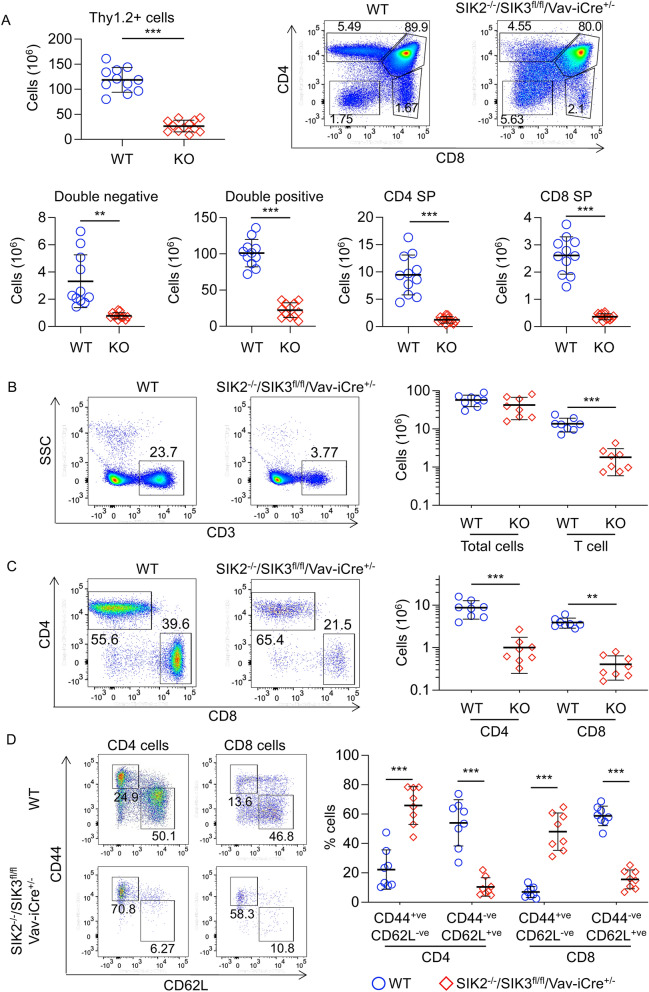
SIK2^-/-^/SIK3^fl/fl^/Vav-iCre^+/-^ mice show abnormal T cell development. (**A**) Thymi were isolated from wild type (WT) and SIK2^-/-^/SIK3^fl/fl^/Vav-iCre^+/-^ (KO) mice and analysed by flow cytometry. Data show the number of Thy1.2^+ve^ cells in the thymus, representative CD4/CD8 flow cytometry plots of Thy1.2^+ve^ cells in the thymus and the numbers of DN, DP, CD4 SP and CD8 SP cells. (**B**–**D**) Splenocytes were isolated from wild type (WT) and SIK2^-/-^/SIK3^fl/fl^/Vav-iCre^+/-^ (KO) mice and analysed by flow cytometry following lysis of red blood cells. Single cell suspensions were analysed for expression of CD3, CD4, CD8, CD44 and CD62L. Total cell numbers and numbers of CD3^+ve^ T cells in the spleen, along with representative FACS plots are shown in (**B**). Absolute numbers of CD4 and CD8 T cells along with representative CD4/CD8 plots of CD3^+ve^ T cells are shown in (**C**). The expression of CD44 and CD62L was also examined in both CD3^+ve^/CD4^+ve^ and CD3^+ve^/CD8^+ve^ T cells and the data shows representative flow cytometry plots and the percentage of CD44^+ve^/CD62L^-ve^ and CD44^-ve^/CD62L^+ve^ cells (**D**). Graphs show mean with symbols representing measurements from individual mice. Differences in cell number between wild type and knockout mice were analysed by Student’s t-test (**B** and Thy1^+ve^ cells in **A**) or RM two way ANOVA with Sidak’s post hoc testing in all other graphs. *p* < 0.05 is indicated by *, < 0.01 by ** and < 0.001 by ***. For the thymus 11 WT and 12 knockout mice were analysed while for the spleens 8 mice were examined per genotype.

Analysis of the thymi from SIK2^-/-^/SIK3^fl/fl^/Vav-iCre^+/-^ mice showed that there was a major decrease in thymocyte number (Fig. 5A). This corresponded to a decrease in the DN, DP and SP subsets (Fig. 5A). Previous studies have not shown a major effect of the Vav-iCre transgene on T cell development, and consistent with the effect seen in Fig. 5A being due to loss of SIK2 and SIK3, a similar drop was not seen in the SIK3^fl/fl^/Vav-iCre^+/−^ mice (see Fig. 2A). To confirm this, a combination of Cre^+ve^ and Cre^−ve^ mice that were wild type for SIK2 and SIK3 were analysed in these experiments. Comparison of these showed that the Vav-iCre transgene was not affecting the numbers of T cell subsets in the thymus (supplementary Fig. 2C). In line with the decreased thymocyte number, the SIK2^-/-^/SIK3^fl/fl^/Vav-iCre^+/-^ mice had greatly reduced numbers of T cells in their spleen (Fig. 5B), which was due to a low number of both CD4 and CD8 T cells (Fig. 5C). Analysis of CD44 and CD62L in the splenic T cells showed that both the CD4 and CD8 cells were enriched in CD44^+ve^/CD62L^−ve^ cells in SIK2^-/-^/SIK3^fl/fl^/Vav-iCre^+/−^ mice (Fig. 5D), suggesting an activated phenotype. This may reflect expansion in the periphery due to the low number of T cells exiting the thymus. Similar results were obtained in the lymph nodes with lower numbers of both CD4 and CD8 cells being present. As in the spleen, the remaining T cells were enriched in CD44^+ve^/CD62L^−ve^ cells (Supplementary Fig. 4). In addition to the number of αβ T cells being reduced, the numbers of γδ T cells were also lower in SIK2^-/-^/SIK3^fl/fl^/Vav-iCre^+/−^ mice (supplementary Fig. 5). In contrast to the effect on T cells, SIK2/3 knockout did not reduce the total numbers of B cells in the spleen or lymph nodes (supplementary Fig. 6). Follicular B cells make up the majority of the B cells in the spleen and the numbers of these cells was not reduced in the SIK2^-/-^/SIK3^fl/fl^/Vav-iCre^+/-^ mice, although there was a trend for a decrease in marginal zone B cells this did not reach significance (*p* > 0.05, supplementary Fig. 6).

**Figure 6:**
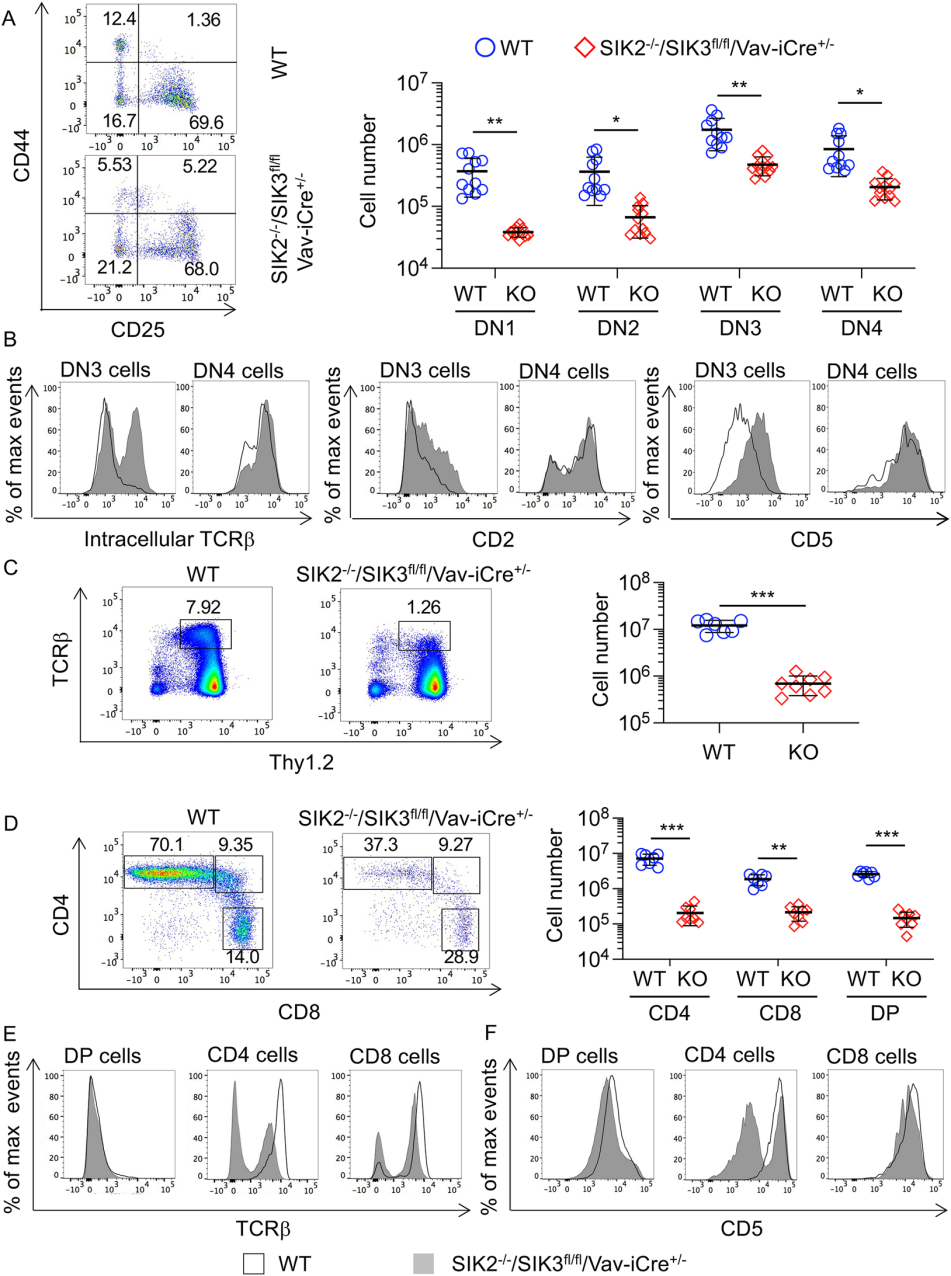
Thymic T cell development is compromised in SIK2^-/-^/SIK3^fl/fl^/Vav-iCre^+/-^ mice. Thymi were isolated from wild type (WT) and SIK2^-/-^/SIK3^fl/fl^/Vav-iCre^+/-^ (KO) mice and analysed by flow cytometry. To identify DN cell subsets, Thy1.2^+ve^ CD4^-ve^ CD8^-ve^ cells were gated and analysed for CD25 and CD44 expression. Representative FACS plots and absolute numbers of the different DN subsets (n = 11 for WT and n = 12 for knockouts) are shown in (**A**). Intracellular staining for TCRβ and cell surface expression of CD2 and CD5 in DN3 (Thy1.2^+ve^ CD4^−ve^ CD8^−ve^ CD44^−ve^ CD25^+ve^) and DN4 (Thy1.2^+ve^ CD4^−ve^ CD8^−ve^ CD44^−ve^ CD25^−ve^) cells is shown in (**B**). Thymocytes were also analysed for expression of cell surface TCRβ. Representative plots of Thy1.2 vs TCRβ in live thymocytes along with quantification of TCRβ^high^ cells is shown in (**C**). Analysis of CD4 and CD8 expression in the TCRβ^high^ cells is shown in (**D**). For C and D, n = 7 for WT and n = 8 for knockouts. Representative histograms for cell surface expression of TCRβ and CD5 in DP, CD4 and CD8 SP cells are shown in (**E** and **F**). Graphs show mean with symbols representing measurements from individual mice. Differences in cell number between wild type and knockout mice were analysed by Student’s t-test. *p* < 0.05 is indicated by *, < 0.01 by ** and < 0.001 by ***.

As the SIK2^−/−^/SIK3^fl/fl^/Vav-iCre^+/−^ mice had reduced DN T cells in the thymus, development of DN cells was examined to determine if SIK2 and SIK3 affected the progression of these cells through development. DN cells can be divided into several stages, DN1 to DN4, based on their expression of CD25 and CD44. The ratios of the different DN subsets were similar between wild type and the SIK2^−/−^/SIK3^fl/fl^/Vav-iCre^+/−^ mice (Fig. 6A), although the absolute numbers were reduced in the SIK2^−/−^/SIK3^fl/fl^/Vav-iCre^+/−^ mice (Fig. 6A). Cells progressing from DN3 to DN4 undergo a process referred to as β-selection, which requires a productive VDJ recombination event at the TCRβ locus and association of the expressed TCRβ chain with pre-Tα to form a pre-TCR^34,35^. Analysis of intracellular levels of the TCRβ chain by flow cytometry showed that SIK2^-/-^/SIK3^fl/fl^/Vav-iCre^+/-^ mice exhibited increased TCRβ levels in DN3 cells relative to SIK2^+/+^/SIK3^+/+^/Vav-iCre^+/-^ controls (Fig. 6B). Signalling downstream of the pre-TCR leads to the upregulation of CD2 and CD5 in DN4 cells relative to DN3 cells^39,40^. Similar to wild type DN cells, the SIK2^-/-^/SIK3^fl/fl^/Vav-iCre^+/-^ DN4 cells expressed higher levels of CD2 and CD5 than DN3 cells (Fig. 6B), indicating that the loss of SIK2 and SIK3 did not prevent the formation of a functional pre-TCR. Interestingly, SIK2^-/-^/SIK3^fl/fl^/Vav-iCre^+/-^ DN3 cells expressed higher levels of CD2 and CD5 than wild type DN3 cells, possibly reflecting the higher expression of intracellular TCRβ in the SIK2^-/-^/SIK3^fl/fl^/Vav-iCre^+/-^ cells (Fig. 6B). Taken together, this data indicated that the loss of SIK2 and SIK3 does not prevent the formation of a functional pre-TCR, although the timing of β-selection may be earlier in the SIK2^-/-^/SIK3^fl/fl^/Vav-iCre^+/-^ cells. SIK2^-/-^/SIK3^fl/fl^/Vav-iCre^+/-^ thymi had an atypical population that was CD8 negative but intermediate for CD4 and not present in the wild type mice (Fig. 5A). The expression of CD2, CD5, CD25, CD44 and intracellular TCRβ was also examined in these cells (supplementary Fig. 7). These cells were CD44^-ve^ and had variable levels of CD25. They also expressed intracellular TCRβ but low levels of cell surface TCRβ. In addition, they expressed CD2 and CD5. Together, these results suggested that they were derived from DN3/4 cells that had undergone rearrangement of the TCRβ chain (supplementary Fig. 7). When these cells were included in an extended double negative gate, the numbers of DN3 and DN4 cells were similar between SIK2^-/-^/SIK3^fl/fl^/Vav-iCre^+/-^ and wild type mice, although DN1 and DN2 cells were still lower (supplementary Fig. 7).

**Figure 7:**
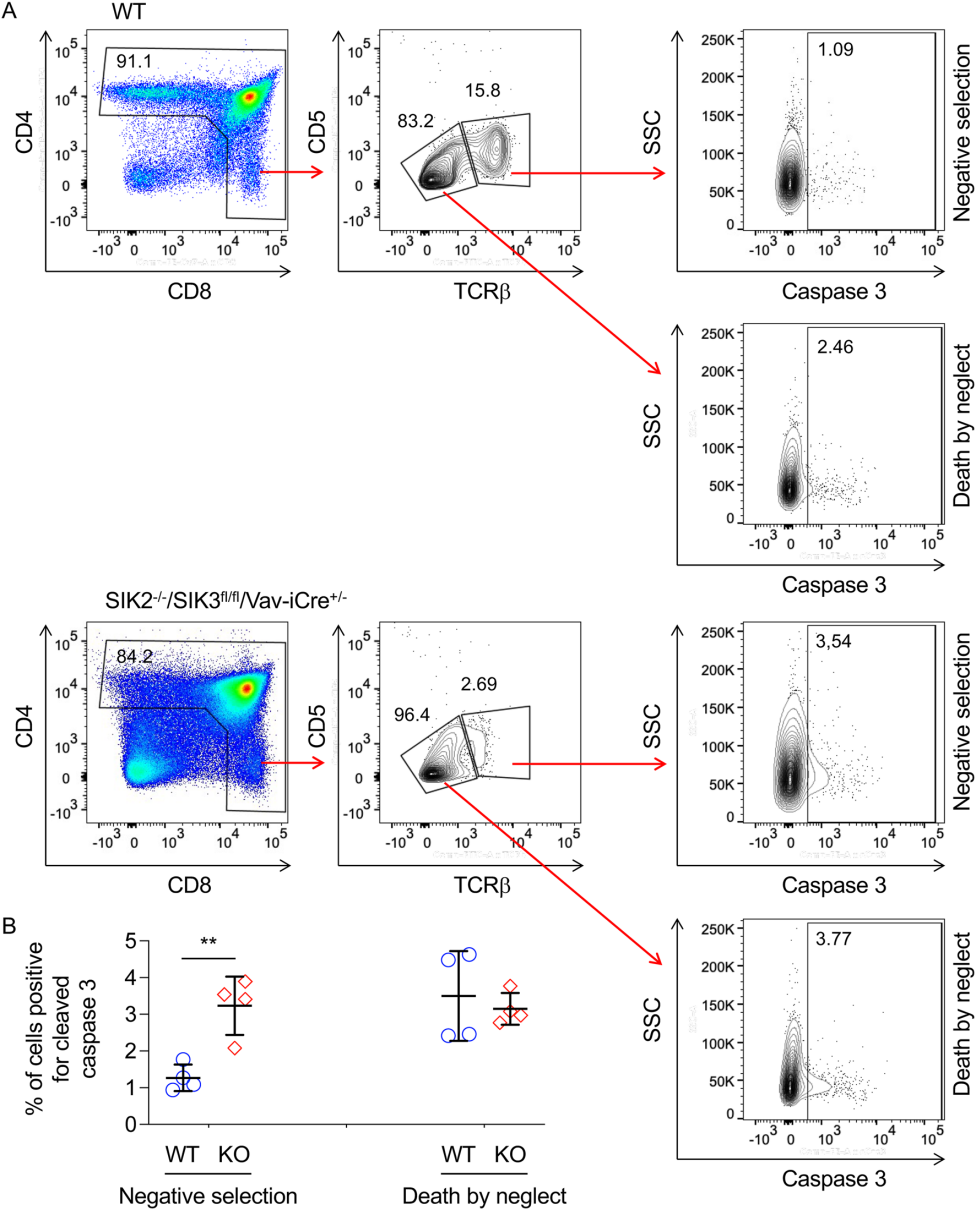
Loss of SIK2 and SIK3 resulted in increased apoptosis in cells undergoing negative selection. Thymi were isolated from wild type and SIK2^−/−^/SIK3^fl/fl^/Vav-iCre^+/−^ (KO) mice and stained for CD4, CD8, TCRβ, CD5 and cleaved caspase 3. Gating on expression of CD4 and/or CD8 was used to identify cells at the DP or SP stage of development. Cells were then gated based on TCRβ and CD5 to identify activated cells that may be undergoing negative selection or DP cells that were TCRβ^low^ and may be undergoing positive selection. The percentage of cells positive for cleaved caspase 3 was then determined in these gates. Representative gating is shown in (**A**), and quantification of 4 mice per genotype in (**B**). Data was analysed by RM two way ANOVA and Sidak’s post hoc testing with ** indicating a *p* value < 0.01.

Following progression to the DP stage, cells rearrange their TCRα gene and start to express a mature TCR on their cell surface^34,35^. These DP cells then undergo positive and negative selection to remove cells with either non-functional or self-reactive T cell receptors. Cells passing selection further upregulate their cell surface TCR levels and transition to SP cells. Notably the fraction of TCR high cells in the thymus was markedly reduced in SIK2^-/-^/SIK3^fl/fl^/Vav-iCre^+/-^ thymi (Fig. 6C). As expected, the majority of the TCR high cells in wild type thymi corresponded to SP cells (Fig. 6D). In SIK2^-/-^/SIK3^fl/fl^/Vav-iCre^+/-^ thymi, the TCR high cells were also mainly SP cells, but their numbers were greatly reduced (Fig. 6D). Although SIK2^-/-^/SIK3^fl/fl^/Vav-iCre^+/-^ DP cells were able to express low levels of TCR on their cell surface, analysis of total CD4 and CD8 SP cells showed that a much lower percentage of cells were TCR high in SIK2^-/-^/SIK3^fl/fl^/Vav-iCre^+/-^ relative to wild type thymi (Fig. 6E). In addition to low expression of cell surface TCR, a large proportion of SIK2^-/-^/SIK3^fl/fl^/Vav-iCre^+/-^ CD4 cells also did not express CD5 (Fig. 6F). Levels of cell surface TCRβ and CD5 can be used to identify activated cells that could be undergoing negative selection. Staining for cleaved caspase 3, which marks cells entering apoptosis has been reported to provide an indication of the numbers of cells undergoing clonal deletion in this population^41^. In the activated T cell population, SIK2^-/-^/SIK3^fl/fl^/Vav-iCre^+/−^ T cells showed an increased percentage of cells positive for cleaved caspase 3 compared to wild type cells, suggesting that there were increased numbers of T cells undergoing clonal deletion during negative selection (Fig. 7). In contrast, the percentage of cells staining positive for cleaved caspase 3 in the TCRβ^low^ gate, where cells may be undergoing positive selection and dying by neglect, was similar between the two genotypes (Fig. 7).

**Figure 8:**
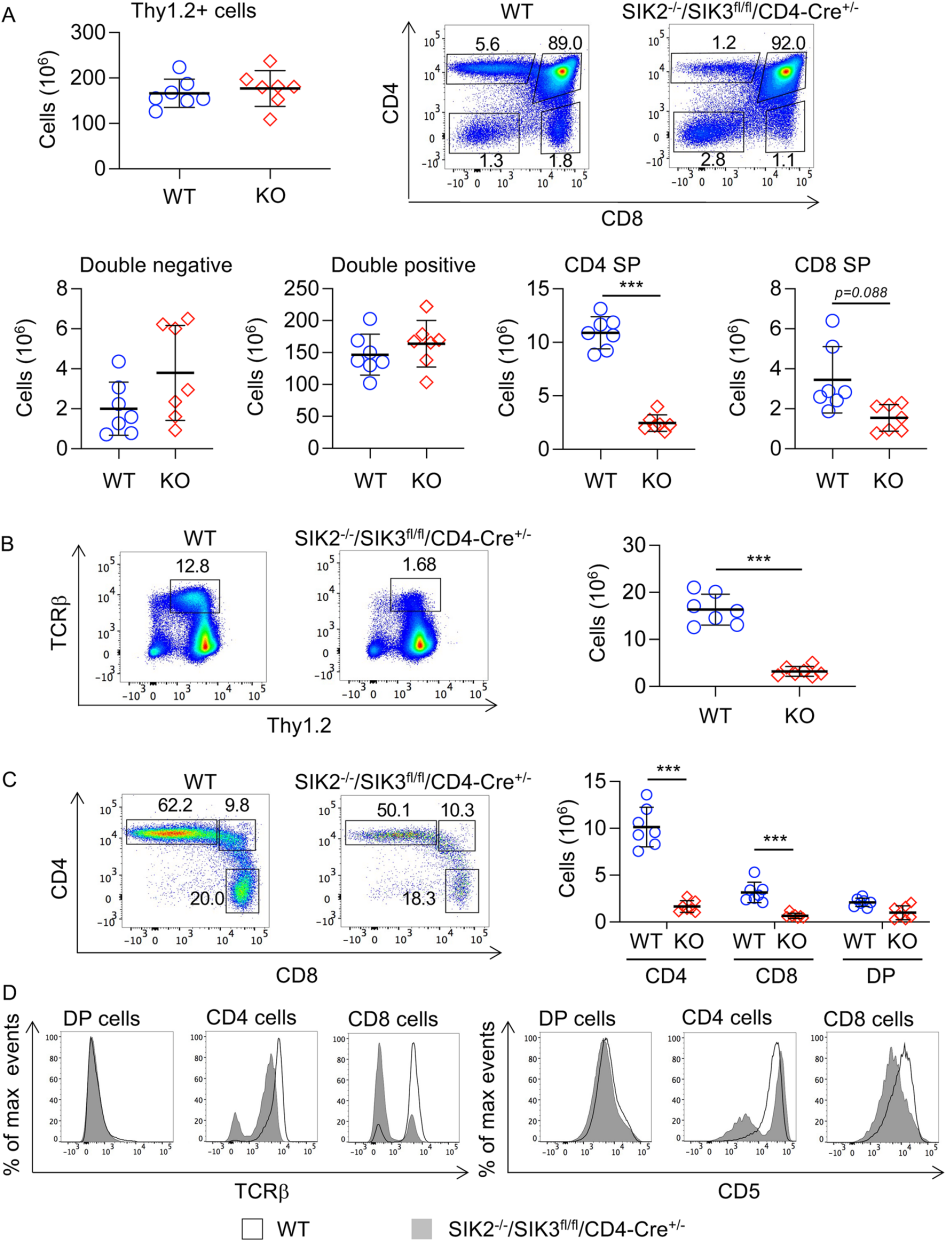
Transition to CD4 and CD8 SP cells is reduced in SIK2^−/−^/SIK3^fl/fl^/CD4-Cre^+/−^ mice. Thymi were isolated from wild type (WT) and SIK2^-/-^/SIK3^fl/fl^/CD4-Cre^+/-^ (KO) mice and analysed by flow cytometry. The number of Thy1.2^+ve^ cells in the thymus along with representative CD4/CD8 plots and numbers of DN, DP and SP cells is shown in (**A**). Thymocytes were also analysed for expression of cell surface TCRβ. Representative plots of Thy1.2 vs TCRβ in live thymocytes along with quantification of TCRβ^high^ cells is shown in (**B**). Analysis of CD4 and CD8 expression in the TCRβ^high^ cells is shown in (**C**). Representative histograms for cell surface expression of TCRβ and CD5 in DP, CD4 and CD8 SP cells are shown in (**D**). Graphs show mean with symbols representing measurements from individual mice. Differences in cell number between wild type and knockout mice were analysed by two tailed Student’s t-test (**A**, **B**) or two way ANOVA with Sidak’s post hoc testing. *p* < 0.05 is indicated by *, < 0.01 by ** and < 0.001 by ***. Graphs show data for 7 mice per genotype.

The above data indicated that in the absence of SIK2 and SIK3, cells cannot progress effectively from DP to SP cells. Vav-iCre however deletes early in haemopoietic development and it is possible that this phenotype is not due to an acute phenotype in DP cells, but a carry-over from issues earlier in T cell development. To further examine the role that SIK2 and SIK3 play in the progression from DP to SP cells, the SIK2^-/-^/SIK3^fl/fl^ mice were crossed with a CD4-Cre transgenic mouse to delete SIK3 at the DP stage of T cell development. The SIK2^-/-^/SIK3^fl/fl^/CD4-Cre^+/-^ mice had similar thymocyte numbers to wild type controls and numbers of DN and DP cells were not decreased in the knockout (Fig. 8A). In contrast, both CD4 and CD8 SP cell numbers were decreased in the SIK2^−/−^/SIK3^fl/fl^/CD4-Cre^+/−^ thymi relative to wild type controls (Fig. 8A). Similar to the SIK2^−/−^/SIK3^fl/fl^/Vav-iCre^+/−^ mice, in the SIK2^−/−^/SIK3^fl/fl^/CD4-Cre^+/−^ thymi the numbers of TCRβ high cells was lower than in wild type thymi, as was the numbers of CD4 and CD8 TCRβ high single positive cells (Figs. 8B, C). Again, similar to SIK2^-/-^/SIK3^fl/fl^/Vav-iCre^+/-^ SP cells, the proportion of SIK2^−/−^/SIK3^fl/fl^/CD4-Cre^+/−^ SP cells expressing high levels of surface TCR was lower than for wild type cells (Fig. 8D). In addition, for those cells that were TCRβ high, the intensity of staining was lower in SIK2^−/−^/SIK3^fl/fl^/CD4-Cre^+/-^ SP cells compare to wild type cells. A proportion of the SIK2^-/-^/SIK3^fl/fl^/CD4-Cre^+/-^ CD4 SP cells also expressed low levels of CD5, but this was not as pronounced as in the SIK2^-/-^/SIK3^fl/fl^/Vav-iCre^+/-^ mice (compare Figs. 6F and 8D).

**Figure 9:**
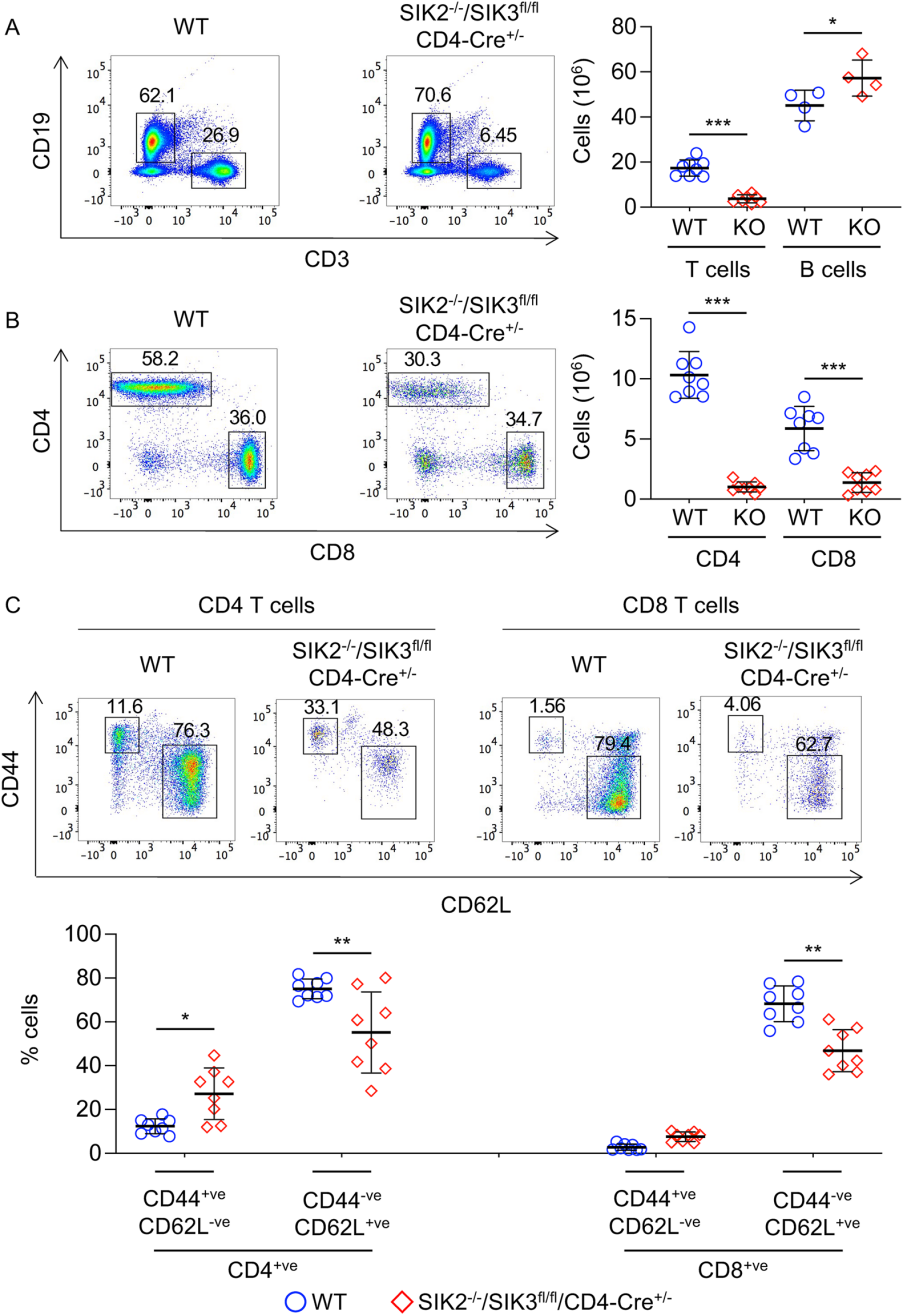
T cell profiles in the spleen of SIK2^−/−^/SIK3^fl/fl^/CD4-Cre^+/-^ mice. Splenocytes were isolated from wild type (WT) and SIK2^−/−^/SIK3^fl/fl^/CD4-Cre^+/-^ (KO) mice and analysed by flow cytometry following lysis of red blood cells. Single cell suspensions were analysed for expression of CD3, CD4, CD8, CD44 and CD62L. A subset of mice were also stained for CD19 to identify B cells. Numbers of CD3^+ve^ T cells and CD19^+ve^ B cells in the spleen, along with representative FACS plots are shown in (**A**). Absolute numbers of CD4 and CD8 T cells along with representative CD4/CD8 plots of CD3^+ve^ T cells are shown in (**B**). The expression of CD44 and CD62L was also examined in both CD3^+ve^/CD4^+ve^ and CD3^+ve^/CD8^+ve^ T cells and data shows representative flow cytometry plots of the percentage of CD44^+ve^/CD62L^-ve^ and CD44^-ve^/CD62L^+ve^ cells (**C**). Graphs show mean and standard deviation and with symbols representing measurements from individual mice (8 mice per genotype for T cells and 4 mice per genotype for B cells). Differences in cell number between wild type and knockout mice were analysed by two tailed Student’s t-test in (A) and RM two way ANOVA and Sidak’s post hoc testing in (B) and (C). *p* < 0.05 is indicated by *, < 0.01 by ** and < 0.001 by ***.

In line with the decrease in SP cells in the thymus, the spleens of SIK2^-/-^/SIK3^fl/fl^/CD4-Cre^+/−^ mice had lower numbers of T cells (Fig. 9A). This drop was more pronounced for the CD4 T cells relative to the CD8 T cells in the spleen (Fig. 9B). Analysis of CD44 and CD62L expression showed that the SIK2^-/-^/SIK3^fl/fl^/CD4-Cre^+/-^ CD4 cells were enriched in CD44^+ve^/CD62L^−ve^ cells (Fig. 9C), although this was not as pronounced as in the SIK2^-/-^/SIK3^fl/fl^/Vav-iCre^+/-^ mice (compare Figs. 9C and 5D). Similar results were obtained for expression of CD44 and CD62L in SIK2^-/-^/SIK3^fl/fl^/CD4-Cre^+/-^ and wild type CD8 T cells in the spleen (Fig. 9C) or when T cells were analysed in the lymph nodes of SIK2^-/-^/SIK3^fl/fl^/CD4-Cre^+/-^ mice (Supplementary Fig. 8).

**Figure 10:**
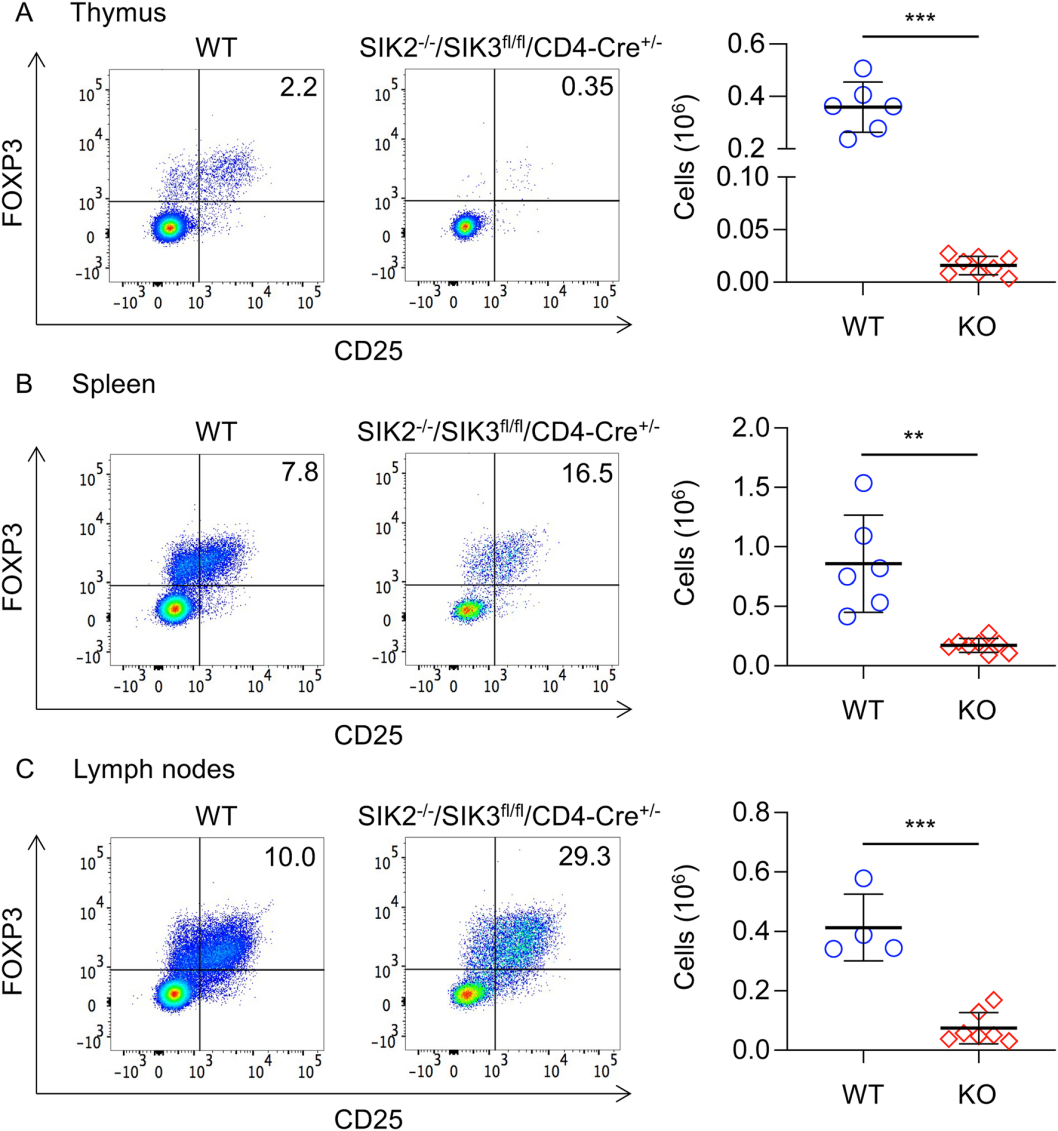
Analysis of Tregs in SIK2^-/-^/SIK3^fl/fl^/CD4-Cre^+/-^ mice. Thymi, spleen and lymph nodes were isolated from wild type (WT) and SIK2^−/−^/SIK3^fl/fl^/CD4-Cre^+/−^ (KO) mice. Cells were stained for TCRβ, CD4, CD25 and FoxP3. Tregs were identified as TCRβ^+ve^/CD4^+ve^/CD25^+ve^/FoxP3^+ve^ cells. Representative CD25 / FoxP3 plots of live gated TCRβ^+ve^/CD4^+ve^ cells are shown along with absolute numbers of Tregs are shown for thymus (**A**), spleen (**B**) and lymph nodes (**C**). Differences in cell number between wild type and knockout mice were analysed by Student’s t-test. *p* < 0.01 by ** and < 0.001 by ***. Data show results from 6 wild type and 8 knockout mice for the thymus and spleen and 4 wild type and 7 knockout mice for the lymph nodes.

CD4^+ve^/CD25^+ve^/FOXP3^+ve^ cells (Tregs) are an important immunosuppressive T cell subset^42^. Analysis of CD4 cells in the thymus, spleen and lymph nodes of SIK2^-/-^/SIK3^fl/fl^/CD4-Cre^+/-^ mice revealed that an increased percentage of the CD4 T cells in the spleen and lymph nodes were Tregs relative to wild type mice (Fig. 10). Despite this, due to the reduced numbers of CD4 T cells in the mice, the absolute numbers of Tregs were lower in the thymus, spleen and lymph nodes of SIK2^−/−^/SIK3^fl/fl^/CD4-Cre^+/−^ relative to wild type mice (Fig. 10). Similar results were obtained in SIK2^−/−^/SIK3^fl/fl^/Vav-iCre^+/−^ mice (supplementary Fig. 9).

## Discussion

The results presented in this paper show that SIK2 and SIK3 have an important role in T cell development. Loss of SIK2 kinase activity alone had little impact on T cell development while loss of SIK3 caused a modest reduction in thymocyte and peripheral T cell numbers. A combined loss of SIK2 and SIK3, however, resulted in a large reduction in T cells in the spleen and lymph nodes and a failure of T cell differentiation in the thymus. This suggests that SIK2 and SIK3 are the major functional SIK isoforms in T cells and that SIK2 can partially compensate for the loss of SIK3. In this study, two Cre transgenes were used to study SIK function in T cells. Vav-iCre, which deletes within immune progenitors prior to the entry of cells into the thymus, and CD4-Cre which deletes at the DP stage of thymic development. As would be expected, deletion via CD4-Cre had a less pronounced effect on early T cell development in the thymus, but did reduce the progression of DP cells to CD4 and CD8 SP cells. Deletion using either Vav-iCre or CD4-Cre resulted in a reduction of TCRβ^hi^ DP cells and a decreased progression of cells to the SP stage. The greatly reduced numbers of TCRβ^hi^ CD4 and CD8 SP cells in these mice could indicate an increased rate of clonal deletion during negative selection. Consistent with this SIK2^-/-^/SIK3^fl/fl^/Vav-iCre^+/-^ TCRβ^hi^/CD5^+ve^ cells, which are likely to be undergoing selection, showed a higher rate of apoptosis than the equivalent wild type population. This does not however exclude the possibility that SIKs may also play additional roles in positive selection. SIK2^−/−^/SIK3^fl/fl^/Vav-iCre^+/−^ mice also showed a reduction in the numbers of DP cells, potentially suggesting that SIK2 and SIK3 play a role earlier in T cell development, in addition to their effects on the DP to SP transition. One reason for a block in thymic development can be a failure to rearrange the TCRβ or TCRα locus at the DN3 and DP stage of development. Loss of SIK2 and SIK3 however did not prevent TCR chain rearrangement, as evidenced by expression of an intracellular TCRβ chain in DN3 and DN4 cells, as well as the upregulation of CD2 and CD5 in DN4 cells. In fact, loss of SIK2 and SIK3 resulted in a higher expression of intracellular TCRβ in DN3 cells, suggesting that TCRβ rearrangement might be promoted rather than inhibited by deletion of SIK2 and SIK3. Furthermore, the initial low-level expression of TCR on the surface of DP cells was unaffected by loss of SIK2 and SIK3, suggesting that TCRα chain rearrangement was able to occur. It is however possible that, as discussed below, the loss of SIK2 and SIK3 may affect TCR chain diversity following VDJ recombination.

The molecular mechanism by which SIK2 and SIK3 regulate T cell development requires further study. For SIKs to be active, they must be constitutively phosphorylated by LKB1^10^. Conditional knockout of LKB1 early in T cell development using Lck-Cre did not prevent expression of the pre-TCR but did block progression from DN to DP cells^43^. In addition to SIKs, LKB1 is also necessary for the activity of a number of other members of the AMPK family of protein kinases, which could explain the earlier block observed upon loss of LKB1 compared to loss of SIK2 and SIK3. Knockout of LKB1 driven via CD4-Cre resulted in cells that failed to progress efficiently from DP to SP cells^44^, similar to what was observed for double knockout of SIK2 and SIK3 in this study. SIKs are able to phosphorylate CRTCs and thus inhibit their ability to function as co-activator proteins for CREB. The effect of CRTC1 and CRTC3 knockout on T cells has not been reported, while CRTC2 is required for the efficient generation of Th17 cells but not for T cell development in the thymus^27^. At present, the only established target for CRTCs is the transcription factor CREB, which can be activated by phosphorylation on Ser133 and/or by the recruitment of CRTCs to its bZIP domain. However, CREB function is not essential for T cell development in adult mice. Conditional knockout of CREB using Lck-Cre in mice lacking ATF1 (a transcription factor closely related to CREB) has been reported. This demonstrated that lack of CREB and ATF1 did not block thymic T cell development although there was a decrease in thymocyte number in the double knockout mice^45^. Significantly, CD2, CD5 and TCRβ expression were normal and T cells were present in the periphery, in contrast to the SIK2/SIK3 double knockout mice studied here. Blocking TCR-induced CREB phosphorylation, either via knockout of both MSK1 and MSK2^32^ or by mutation of the codon for Ser133 in the CREB gene (unpublished observations) also does not suppress T cell development in adult mice. However, it is noteworthy that CREB has been reported to be required for normal development of the embryonic thymus. The knockout of CREB reduced the number of TCRβ^high^ cells^46^, while overexpression of ICER (an inhibitor of CREB mediated transcription) decreased cell number and delayed progression to DP cells in foetal thymic organ cultures^47^. In our model, the loss of both SIK2 and SIK3 would be expected to increase rather than block the induction of CREB-dependent genes so it is possible that some of the observed phenotype arises from an increased activation of CREB in developing thymocytes.

Another set of substrates for SIKs are class IIa HDACs, a family that includes HDACs 4, 5, 7 and 9. The individual knockout of HDAC4 or HDAC5 does not compromise thymic T cell development, although HDAC5^-/-^ Tregs were reported to have less suppressive activity^48,49^. A detailed analysis of HDAC9 deficiency has not been published, but HDAC9^-/-^ mice do have peripheral T cells^50^. HDAC9 is, however, important in Tregs and HDAC9^-/-^ Tregs show enhanced suppressive activity relative to wild type Tregs^50^. HDAC7 is highly expressed in the thymus^51^ and like SIK3 is upregulated as cells progress to the DP stage of development (supplementary Fig. 1). Conditional knockout of HDAC7 driven via Lck-Cre results in a strong reduction of CD4 and CD8 SP cells in the thymus along with a more moderate reduction in DP cells. This was attributed to increased apoptosis of the HDAC7^-/-^ cells, resulting in a failure to establish a full repertoire of TCRα rearrangements leading to impaired positive selection^52^. This has parallels to the SIK2/3 phenotype reported here, although loss of SIK2 and SIK3 results in a stronger reduction in DP cells than in the HDAC7 knockout. SIKs target multiple class IIa HDACs and it is possible that different class IIa HDACs may compensate for each other. Therefore, knockout of multiple HDAC isoforms may give a more severe thymic phenotype. It should also be noted that the phosphorylation of class IIa HDACs by SIKs affects HDAC localisation, but does not necessarily inhibit HDAC function. Thus Ser to Ala knock-in mutations of the sites in class IIa HDACs phosphorylated by SIKs would be required to determine if HDACs were the primary substrate downstream of SIKs in regulating T cell development, or if SIKs also target other substrates in T cells.

In summary we show here that loss of SIK3 results in lower numbers of T cells in secondary lymphoid organs and that combined loss of SIK2 and SIK3 inhibits T cell development in the thymus.

## Methods

### Mice

The generation of kinase-inactive SIK1 (SIK1^ki/ki^; gene symbol SIK1^tm1.1Arte^), SIK2 (SIK2^ki/ki^; gene symbol SIK2^tm1.1Arte^) and SIK3 (SIK3^ki/ki^; gene symbol SIK3^tm1.1Arte^) knock-in mice as well as the CD4-Cre and Vav-iCre transgenic mice have been described previously^9,53,54^. Neither of these two Cre strains was found to have a major impact on thymic T cell development in previous studies^55^. The SIK2 knock-in has LoxP sites around exons 5 to 7 in SIK2 (which contains the T175A mutation in the activation loop of the kinase domain, abolishing kinase activity). To generate SIK2 knockout mice, the SIK2 knock-in mice were crossed to the Cre Deleter strain (C57BL/6-*Gt(ROSA)26Sor*^*tm16(cre)Arte*^*,* Taconic Biosciences). The resulting SIK2 knockout allele lacks the sequence corresponding to amino acids 160 to 316 in the kinase domain of SIK2 and has a frame shift mutation from exon 4 to 8. These mice were bred away from the Cre transgene.

To generate a conditional SIK3 allele, C57BL/6 N-A^tm1Brd^ Sik3^tm1a(EUCOMM)Hmgu/Wtsi^

mice were obtained from the European Mouse Mutant Archive (EMMA) repository. These mice were crossed to Flpe transgenic mice (Taconic Biosciences) to remove the LacZ and selection cassettes, resulting in an allele (SIK3^fl^) with LoxP sites on either side of exon 5. This exon encodes residues 226 to 247 of SIK3 which includes the DFG motif of the kinase domain that is essential for catalytic activity (Supplementary Fig. 2A). Removal of exon 5 would also result in a frame shift mutation from exon 4 to 6.

Both SIK2 and SIK3 are located on chromosome 9 separated by approximately 4.6 Mb. To generate the SIK2/3 double knockout, SIK2^-/-^ and SIK3^fl/fl^ mice were crossed. The resulting SIK2^+/-^/SIK3^+/fl^ mice were crossed to SIK3^fl/fl^ mice until an SIK2^+/-^/SIK3^fl/fl^ mouse was obtained, which would be indicative of a crossover event occurring to generate mice with the mutant SIK2 and SIK3 alleles on the same chromosome.

All lines were maintained by backcrossing with wild type C57Bl6/J mice obtained from Charles River Laboratories UK. The mice were housed in individually ventilated cages at 21 °C, 45–55% humidity, and a 12/12-h light/dark cycle. The mice were provided with free access to food (R&M3) and water and kept under specific pathogen-free conditions. Mice were culled via a rising concentration of CO_2_ and death confirmed by cervical dislocation.

This work was performed under a UK Home Office Project Licence in accordance with UK and EU regulations and approved by the University of Dundee Ethical Review Committee and in line with ARRIVE guidelines.

Mice were genotyped from ear biopsies by PCR. Protocols for genotyping the SIK kinase-inactive knock-in mice were as reported^9^. Routine genotyping of the SIK2 knockout mice was carried out using the primers AAGAGAGTGTGGGACTAACTTGG, CTTAAAAGCTGGGCATAGTGG and TGTTCTCTAAGCATGCTAACTACTAGG, which gives a 395 bp band for a wild type allele and a 557 bp band for a knockout allele. For the conditional SIK3 line, GCTGAAGACGTGGTGTGGCAG and GCAGGTAACATTTCTGCTTCCAGAC were used which give a 377 bp band and 457 bp band for wild type and floxed alleles respectively. For the Vav-iCre mice the primers CTCCAACCTGCTGACTGTGC and CACCAGGGACACAGCATTGG were used which gives a 350 bp band for Cre^+ve^ animals. For the CD4-Cre mice the primers CAGATTCCCAACCAACAAGAGCTCAAGG and CCCAAATGTTGCTGGATAGTTTTTACTGCC were used which gives a 333 bp band for Cre^+ve^ animals, alongside AAAGTCGCTCTGAGTTGTTAT and GGAGCGGGAGAAATGGATATG to give a 602 bp control band.

To maintain Cre lines as heterozygous animals, male and female Cre^+ve^ mice were not bred together. Due to the possibility of germline deletion in male Vav-iCre^+ve^ mice, Vav-iCre^+ve^ males were not used for breeding^56^. SIK3^fl/fl^/Vav-iCre^+/−^ female mice showed greatly reduced fertility and so SIK3^+/fl^/Vav-iCre^+/−^ mice were used for breeding.

### Flow cytometry

The thymus, spleen and lymph nodes were dissected from wild type and transgenic mice and cells were isolated by pressing the corresponding tissue through a 40 μm EASYstrainer (Greiner Bio-One). For spleens, red blood cells in the resulting cell suspensions were removed using Red Blood Cell Lysing Buffer (Sigma), after which cells were washed and resuspended in FACS buffer (PBS, 1% (w/v) BSA). Viable cells from different tissues were then counted after addition of DAPI (1.25 μg/mL; BioLegend). Cells were counted on a BD FACSVerse.

To avoid non-specific binding of antibodies to Fc receptors, between 2 and 4 × 10^6^ cells were blocked with rat anti-mouse CD16/CD32 Fc block (clone 2.4G2; BD Pharmingen; 1:50 in FACS buffer) for 20 min at 4 °C. Cells were then stained with fluorophore-conjugated antibodies to the required antigens. The antibodies used are listed in Table 1. Flow cytometry data was acquired on a BD FACSCanto II and analysed using FlowJo Version 10 software. Live lymphocytes were gated based on SSC-A and FSC-A and then doublets excluded based on FSC-W / FSC-A plots. For all experiments except the analysis of CD44 and CD62L and cleaved caspase 3, live cells were then gated based on DAPI exclusion.

**Table 1:** Flow cytometry antibodies.

Antibody	Supplier	Fluorophore	Clone number	Dilution
Anti-Thy1.2	BioLegend	APC	53–2.1	1:200
Anti-CD4	BioLegend	PerCP-Cy5.5	GK1.5	1:200
Anti-CD8	BioLegend	PE/Cy-7	53–6.7	1:200
Anti-CD3ε	BioLegend	FITC	145–2C11	1:50
Anti-CD19	BD Pharmingen	APC/Cy7	1D3	1:100
Anti-CD44	BD pharmingen	PE	IM7	1:100
Anti-CD62L	BioLegend	BV421	MEL-14	1:100
Anti-TCRβ	BioLegend	FITC	H57-597	1:50
Anti-CD25	BioLegend	APC/Cy7	PC61	1:100
Anti-CD2	BioLegend	PE	RM2-5	1:100
Anti-CD5	eBiosciences	PE/Cy5	53–7.3	1:100
Anti-CD5	Biolegend	APC	53–7.3	1:50
Anti-FoxP3	eBiosciences	PE	FJK-16 s	1:100
Anti-CD21	BD Pharmingen	FITC	7G6	1:200
Anti-IgM	Biolegend	PerCP-Cy5.5	RMM-1	1:300
Anti-IgM	BD Pharmingen	APC	II/41	1:200
Anti-Cleaved caspase 3	Cell signaling technology	PE	D3E9	1:50

For intracellular TCRβ staining*,* cells were isolated from the thymus as described above. Thymocytes were stained for cell surface markers (with anti-Thy1.2, anti-CD4, anti-CD8, anti-CD44 and anti-CD25) to determine Double Negative (DN) 3 and DN4 cell populations. Cells were then fixed using fixation buffer (eBiosciences) for 20 min at 4 °C, washed with FACS buffer, permeabilized for 20 min at 4 °C in 1 X permeabilization buffer (eBiosciences) and washed with FACS buffer. Cells were then incubated with 1:50 Fc block made up in 1 X permeabilization buffer for 10 min at 4 °C, washed with FACS buffer, and incubated with 1:50 anti-TCRβ antibody made up in 1X permeabilization buffer for 30 min at 4 °C. For staining of Foxp3, the Foxp3/Transcription factor staining buffer set (00–5523-00) from eBioscience was used according to the manufacturer’s instructions. Cleaved caspase 3 was assessed as detailed previously^41^.

To analyse embryonic T cells, matings were checked daily for plugs and embryos isolated at E17.5. The embryonic thymus was isolated and cells stained for Thy1.2, TCRβ, CD4 and CD8 and analysed by flow cytometry as described above. A sample of tissue was retained from each embryo and used for genotyping.

### Immunoblotting

Splenocytes were isolated as above and, following removal of red blood cells, they were lysed as described^9^. Cell extracts were clarified by centrifugation and protein concentrations determined using the Bradford assay. 5 (Fig. 1) or 25 (Supplementary Fig. 2) μg of protein extract was separated by SDS-PAGE and immunoblotted using antibodies described previously^9^. The SIK2 and SIK3 antibodies were obtained from MRC PPU Reagents and Services (https://mrcppureagents.dundee.ac.uk)^9^ and detected using a horseradish peroxidase-conjugated anti-sheep IgG secondary antibody (Abcam). Class IIa HDAC, p38 and GAPDH antibodies were from Cell Signaling Technology and the CRTC3 antibody (clone EPR3440) was from Abcam. The expression of proteins was visualized using a Chemidoc MP imaging system (Bio-Rad Laboratories) or Li-Cor Odyssey Fc following addition of an enhanced chemiluminescence substrate (Amersham).

### Statistical analysis

Numerical data was compiled and processed in Excel. Statistical analysis was carried out in Graphpad Prism, which was also used to generate the graphs. Data shown in Figs. 2, 3, 7 and 8 represent pooled data from two experiments while Figs. 5 and 6 contain data pooled from 3 experiments. Each experiment contained similar numbers of wild type and knockin animals. Data was not pooed from multiple experiments for Figs. 1 and 4. Data is presented as mean and standard deviation with symbols representing individual mice. Significance between the wild type and SIK knockout conditions was assessed by either un-paired two tailed Student’s t-test or repeated measures (RM) two-way ANOVA with Sidak’s post hoc testing as indicated in the figure legends.

## Supplementary Information

Supplementary Information.

## References

[1] Anderson MK (2006). At the crossroads: Diverse roles of early thymocyte transcriptional regulators. Immunol. Rev..

[2] Cantrell DA (2002). Transgenic analysis of thymocyte signal transduction. Nat. Rev. Immunol..

[3] Rothenberg EV, Taghon T (2005). Molecular genetics of T cell development. Ann. Rev. Immunol..

[4] Clark K (2014). Protein kinase networks that limit TLR signalling. Biochem. Soc. Trans..

[5] Altarejos JY, Montminy M (2011). CREB and the CRTC co-activators: Sensors for hormonal and metabolic signals. Nat. Rev. Mol. Cell Biol..

[6] Darling NJ, Cohen P (2021). Nuts and bolts of the salt-inducible kinases (SIKs). Biochem. J..

[7] Wang Z, Takemori H, Halder SK, Nonaka Y, Okamoto M (1999). Cloning of a novel kinase (SIK) of the SNF1/AMPK family from high salt diet-treated rat adrenal. FEBS Lett..

[8] Bright NJ, Thornton C, Carling D (2009). The regulation and function of mammalian AMPK-related kinases. Acta Physiol. (Oxf).

[9] Darling NJ, Toth R, Arthur JS, Clark K (2017). Inhibition of SIK2 and SIK3 during differentiation enhances the anti-inflammatory phenotype of macrophages. Biochem. J..

[10] Lizcano JM (2004). LKB1 is a master kinase that activates 13 kinases of the AMPK subfamily, including MARK/PAR-1. EMBO J..

[11] MacKenzie KF (2013). PGE(2) induces macrophage IL-10 production and a regulatory-like phenotype via a protein kinase A-SIK-CRTC3 pathway. J. Immunol..

[12] Henriksson E (2012). The AMPK-related kinase SIK2 is regulated by cAMP via phosphorylation at Ser358 in adipocytes. Biochem. J..

[13] Patel K (2014). The LKB1-salt-inducible kinase pathway functions as a key gluconeogenic suppressor in the liver. Nat. Commun..

[14] Screaton RA (2004). The CREB coactivator TORC2 functions as a calcium- and cAMP-sensitive coincidence detector. Cell.

[15] Conkright MD (2003). TORCs: Transducers of regulated CREB activity. Mol Cell.

[16] Luo Q (2012). Mechanism of CREB recognition and coactivation by the CREB-regulated transcriptional coactivator CRTC2. Proc. Natl. Acad. Sci. USA.

[17] Clark K (2012). Phosphorylation of CRTC3 by the salt-inducible kinases controls the interconversion of classically activated and regulatory macrophages. Proc. Natl. Acad. Sci. USA.

[18] Bittinger MA (2004). Activation of cAMP response element-mediated gene expression by regulated nuclear transport of TORC proteins. Curr. Biol..

[19] Berdeaux R (2007). SIK1 is a class II HDAC kinase that promotes survival of skeletal myocytes. Nat. Med..

[20] Walkinshaw DR (2013). The tumor suppressor kinase LKB1 activates the downstream kinases SIK2 and SIK3 to stimulate nuclear export of class IIa histone deacetylases. J. Biol. Chem..

[21] Sundberg TB (2014). Small-molecule screening identifies inhibition of salt-inducible kinases as a therapeutic strategy to enhance immunoregulatory functions of dendritic cells. Proc. Natl. Acad. Sci. USA.

[22] Luan B (2015). CREB pathway links PGE2 signaling with macrophage polarization. Proc. Natl. Acad. Sci. USA.

[23] Lombardi MS, Gillieron C, Dietrich D, Gabay C (2016). SIK inhibition in human myeloid cells modulates TLR and IL-1R signaling and induces an anti-inflammatory phenotype. J. Leukoc. Biol..

[24] Elcombe SE (2013). Dectin-1 regulates IL-10 production via a MSK1/2 and CREB dependent pathway and promotes the induction of regulatory macrophage markers. PLoS ONE.

[25] Ananieva O (2008). The kinases MSK1 and MSK2 act as negative regulators of Toll-like receptor signaling. Nat. Immunol..

[26] Sutavani RV (2018). Differential control of Toll-like receptor 4-induced interleukin-10 induction in macrophages and B cells reveals a role for p90 ribosomal S6 kinases. J. Biol. Chem..

[27] Hernandez JB (2015). The CREB/CRTC2 pathway modulates autoimmune disease by promoting Th17 differentiation. Nat. Commun..

[28] Flamand L, Romerio F, Reitz MS, Gallo RC (1998). CD4 promoter transactivation by human herpesvirus 6. J Virol.

[29] Gao MH, Kavathas PB (1993). Functional importance of the cyclic AMP response element-like decamer motif in the CD8 alpha promoter. J. Immunol..

[30] Mayall TP, Sheridan PL, Montminy MR, Jones KA (1997). Distinct roles for P-CREB and LEF-1 in TCR alpha enhancer assembly and activation on chromatin templates in vitro. Genes. Dev..

[31] Anderson SJ, Miyake S, Loh DY (1989). Transcription from a murine T-cell receptor V beta promoter depends on a conserved decamer motif similar to the cyclic AMP response element. Mol. Cell Biol..

[32] Kaiser M, Wiggin GR, Lightfoot K, Arthur JS, Macdonald A (2007). MSK regulate TCR-induced CREB phosphorylation but not immediate early gene transcription. Eur. J. Immunol..

[33] Howden AJM (2019). Quantitative analysis of T cell proteomes and environmental sensors during T cell differentiation. Nat. Immunol..

[34] Starr TK, Jameson SC, Hogquist KA (2003). Positive and negative selection of T cells. Ann. Rev. Immunol..

[35] Naito T, Tanaka H, Naoe Y, Taniuchi I (2011). Transcriptional control of T-cell development. Int. Immunol..

[36] Heng, T. S. P., Painter, M. P. The Immunological Genome Project: networks of gene expression in immune cells. *Nat. Immunol.* **9**, 1091–1094 (2008). 10.1038/ni1008-1091 18800157

[37] Park JE (2020). A cell atlas of human thymic development defines T cell repertoire formation. Science.

[38] Sasagawa S (2012). SIK3 is essential for chondrocyte hypertrophy during skeletal development in mice. Development.

[39] Azzam HS (1998). CD5 expression is developmentally regulated by T cell receptor (TCR) signals and TCR avidity. J. Exp. Med..

[40] Rodewald HR (1993). Fc gamma RII/III and CD2 expression mark distinct subpopulations of immature CD4-CD8- murine thymocytes: In vivo developmental kinetics and T cell receptor beta chain rearrangement status. J. Exp. Med..

[41] Breed ER, Watanabe M, Hogquist KA (2019). Measuring thymic clonal deletion at the population level. J. Immunol..

[42] Josefowicz SZ, Lu LF, Rudensky AY (2012). Regulatory T cells: Mechanisms of differentiation and function. Ann. Rev. Immunol..

[43] Tamas P (2010). LKB1 is essential for the proliferation of T-cell progenitors and mature peripheral T cells. Eur. J. Immunol..

[44] Zarrouk M, Rolf J, Cantrell DA (2013). LKB1 mediates the development of conventional and innate T cells via AMP-dependent kinase autonomous pathways. PLoS ONE.

[45] Baumann S (2004). CREB function is required for normal thymic cellularity and post-irradiation recovery. Eur. J. Immunol..

[46] Rudolph D (1998). Impaired fetal T cell development and perinatal lethality in mice lacking the cAMP response element binding protein. Proc. Natl. Acad. Sci. USA.

[47] Grady GC, Mason SM, Stephen J, Zuniga-Pflucker JC, Michie AM (2004). Cyclic adenosine 5'-monophosphate response element binding protein plays a central role in mediating proliferation and differentiation downstream of the pre-TCR complex in developing thymocytes. J. Immunol..

[48] Liu Q (2017). HDAC4 is expressed on multiple T cell lineages but dispensable for their development and function. Oncotarget.

[49] Xiao H (2016). HDAC5 controls the functions of Foxp3(+) T-regulatory and CD8(+) T cells. Int. J. Cancer.

[50] Tao R (2007). Deacetylase inhibition promotes the generation and function of regulatory T cells. Nat. Med..

[51] Dequiedt F (2003). HDAC7, a thymus-specific class II histone deacetylase, regulates Nur77 transcription and TCR-mediated apoptosis. Immunity.

[52] Kasler HG (2011). Histone deacetylase 7 regulates cell survival and TCR signaling in CD4/CD8 double-positive thymocytes. J. Immunol..

[53] Lee PP (2001). A critical role for Dnmt1 and DNA methylation in T cell development, function, and survival. Immunity.

[54] de Boer J (2003). Transgenic mice with hematopoietic and lymphoid specific expression of Cre. Eur. J. Immunol..

[55] Ananieva O (2008). ERK5 regulation in naive T-cell activation and survival. Eur. J. Immunol..

[56] Siegemund S, Shepherd J, Xiao C, Sauer K (2015). hCD2-iCre and Vav-iCre mediated gene recombination patterns in murine hematopoietic cells. PLoS ONE.

