# Supplementary material for: Salt inducible kinases 2 and 3 are required for thymic T cell development
Source: Sci Rep. 2021 Nov 3;11:21550. doi: 10.1038/s41598-021-00986-0 (PMC8566462; doi:10.1038/s41598-021-00986-0)
Supplement: Supplementary file 1 — Supplementary Information. [file 41598_2021_986_MOESM1_ESM.pdf]

Supplementary Information

Salt Inducible kinases 2 and 3 are required for thymic T cell development.

Meriam Nefla[1,2], Nicola J. Darling[2], Manuel Van Gijsel Bonnello[1,2], Philip Cohen[2] and J. Simon C. Arthur[1]

1 Division of Cell Signalling and Immunology, Wellcome Trust Building, School of Life Sciences, University of Dundee, DD1 5EH, U.K.

2 MRC Protein Phosphorylation and Ubiquitylation Unit, Sir James Black Centre, School of Life Sciences, University of Dundee, Dundee DD1 5EH, U.K.

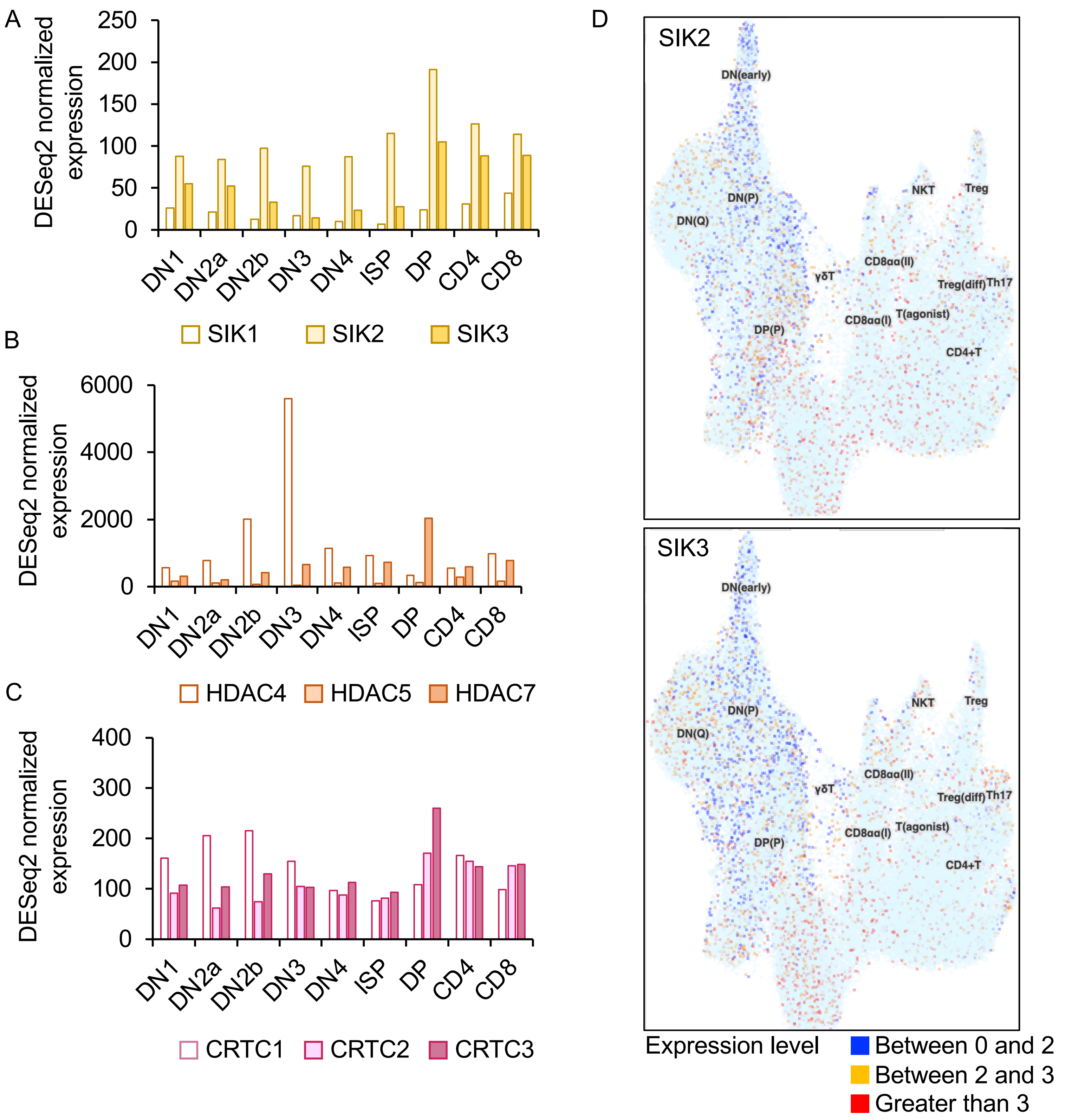

Supplemental Figure 1. SIK levels in CD4 and CD8 T cells.
(A) RNAseq expression data from Immgen (www.immgen.org) showing SIK mRNA expression during thymic T cell development in double negative (DN), intermediate single positive (ISP), CD4/CD8 double positive (DP) and CD4 and CD8 single positive cells. (B) As (A) but showing class IIa HDAC expression. (C) As (A) but showing CRTC expression. (D) SIK2 and SIK3 expression in human thymic subsets from the Human Cell Atlas (https://data.humancellatlas.org/explore/projects/c1810dbc-16d2-45c3-b45e-3e675f88d87b) visualized via the UCSC Cell Browser.

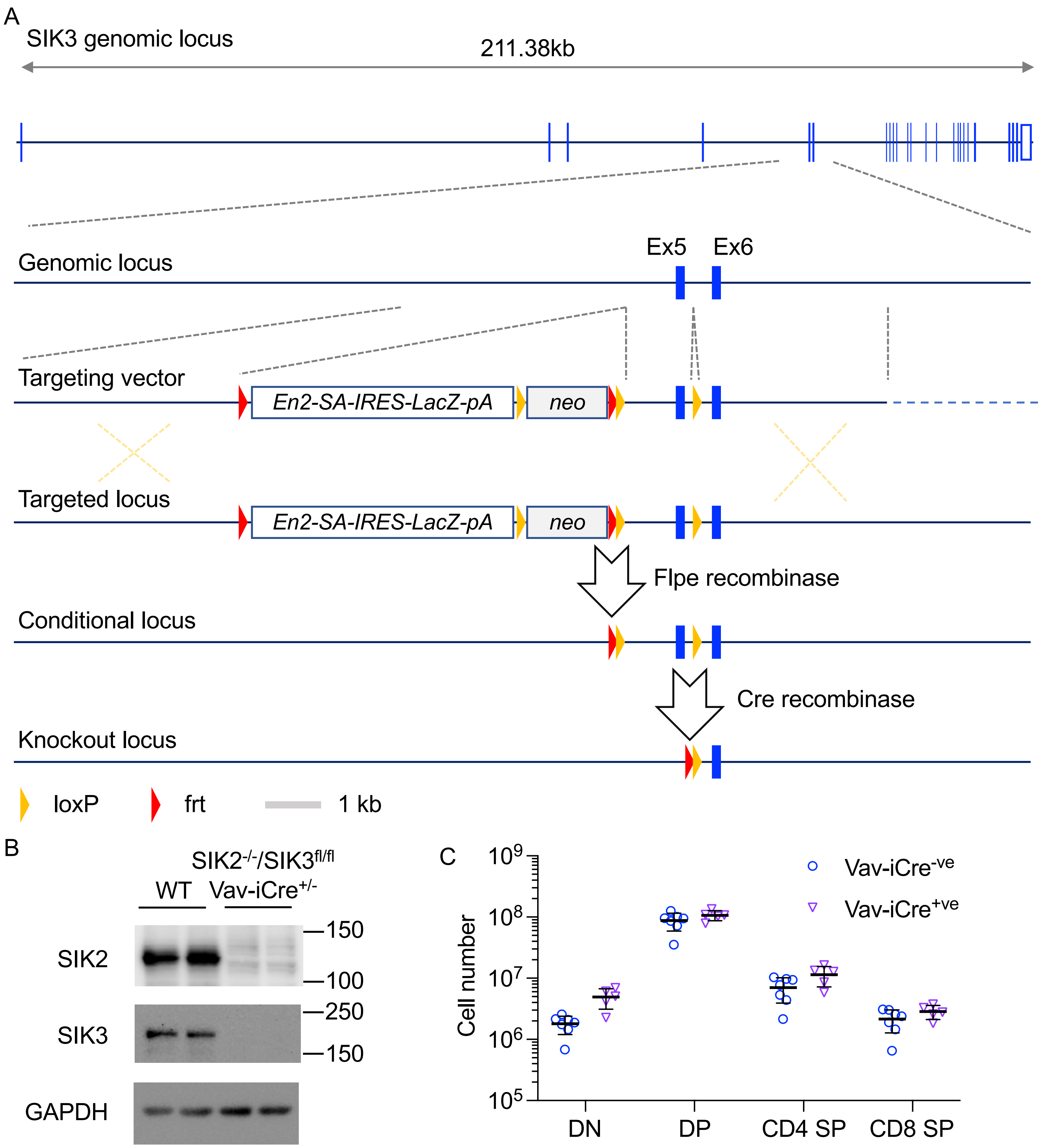

Supplemental Figure 2. Generation of conditional *SIK3* allele.
(A) Schematic of the strategy used to generate the conditional *SIK3* allele. The SIK3 genomic locus is shown (top) along with the targeting vector used to insert Lox P sites either side of exon 5 along with a *LacZ* reporter and neomycin resistance (*neo*) gene flanked by Frt sites. The *LacZ* and *neo* genes were removed by crossing to *Flpe* recombinase transgenic mice to generate mice with a floxed exon 5 in the *SIK3* gene. Final deletion of exon 5 was obtained by crossing to tissue specific Cre transgenics. (B) Immunoblots showing the expression level of SIK2, SIK3 and GAPDH in the spleens of two wild type (WT) and two $SIK2^{-/-}/SIK3^{fl/fl}/Vav\text{-}iCre^{+/-}$ mice. (C) The numbers of DN, DP, CD4 SP and CD8 SP cells in the thymi of mice wild type for SIK2 and 3 but either $Vav\text{-}iCre^{+ve}$ (n=7) or $Vav\text{-}iCre^{-ve}$ (n=5) was determined as described in the methods. No significant effects of the Vav-iCre transgene were seen ($p>0.05$, RM two way ANOVA and Sidak's post hoc testing) .

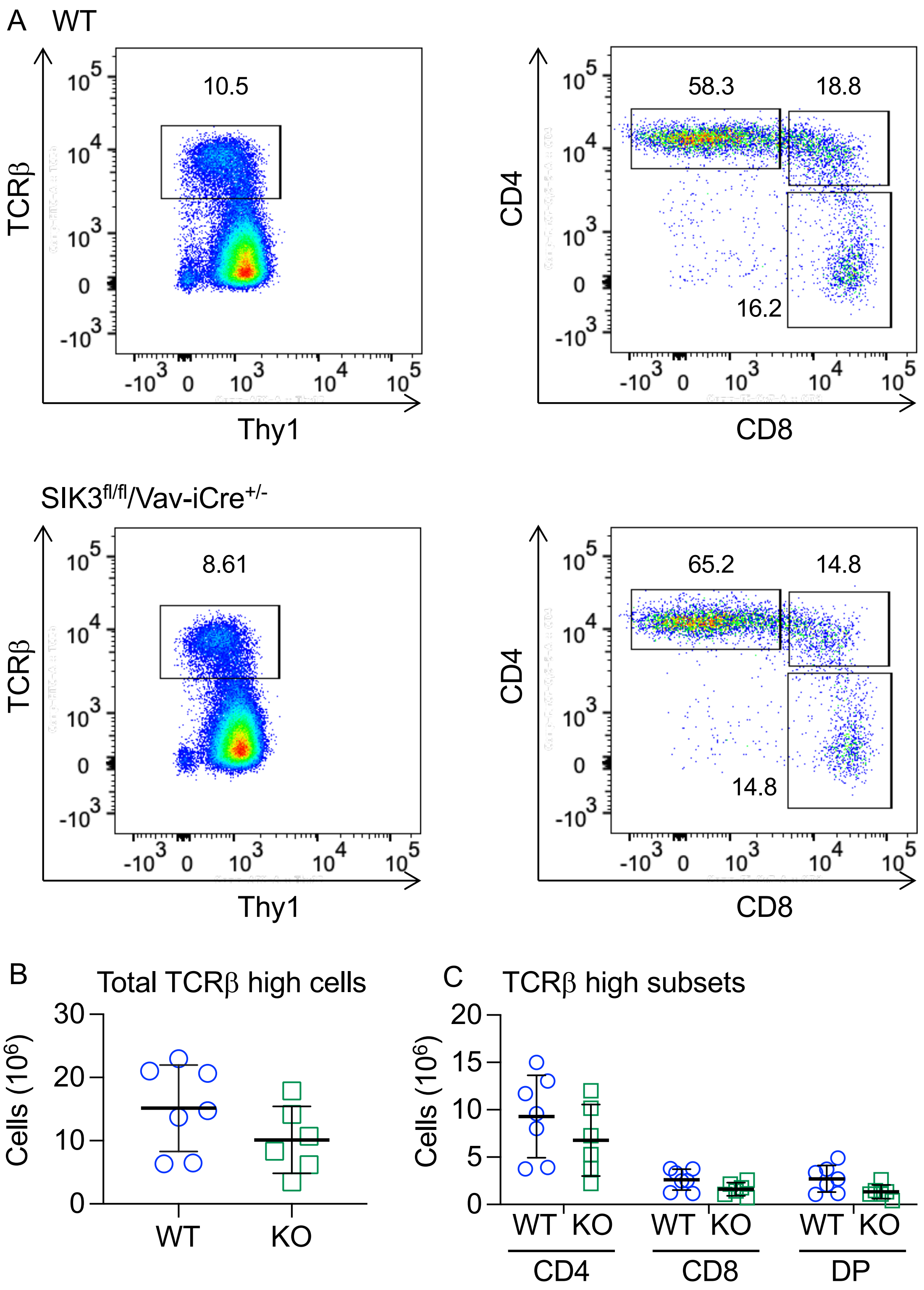

Supplemental Figure 3. Analysis of TCRβ^{high} cells in SIK3$^{fl/fl}$/Vav-iCre$^{+/-}$ thymi.
Thymi were isolated from wild type (WT, n=7) and SIK3$^{fl/fl}$/Vav-iCre$^{+/-}$ (KO, n=6) mice and analysed by flow cytometry. Cells were gated for expression of Thy1 and high levels of cell surface TCRβ (A, left hand plots). Levels of CD4 and CD8 expression in the Thy1+^{ve}/TCRβ^{hi} cells are shown in the right hand panels. The total number of Thy1+^{ve}/TCRβ^{hi} cells is shown in B and the numbers of DP, CD4 SP and CD8 SP cells that were also Thy1+^{ve}/TCRβ^{hi} is shown in C. Graphs show mean and standard deviation with individual mice indicated by symbols. Differences between wild type and knockout mice were analysed by Student's t-test (B) or RM two way ANOVA and Sidak's post hoc testing (C). Significant differences (p < 0.05) were not identified.

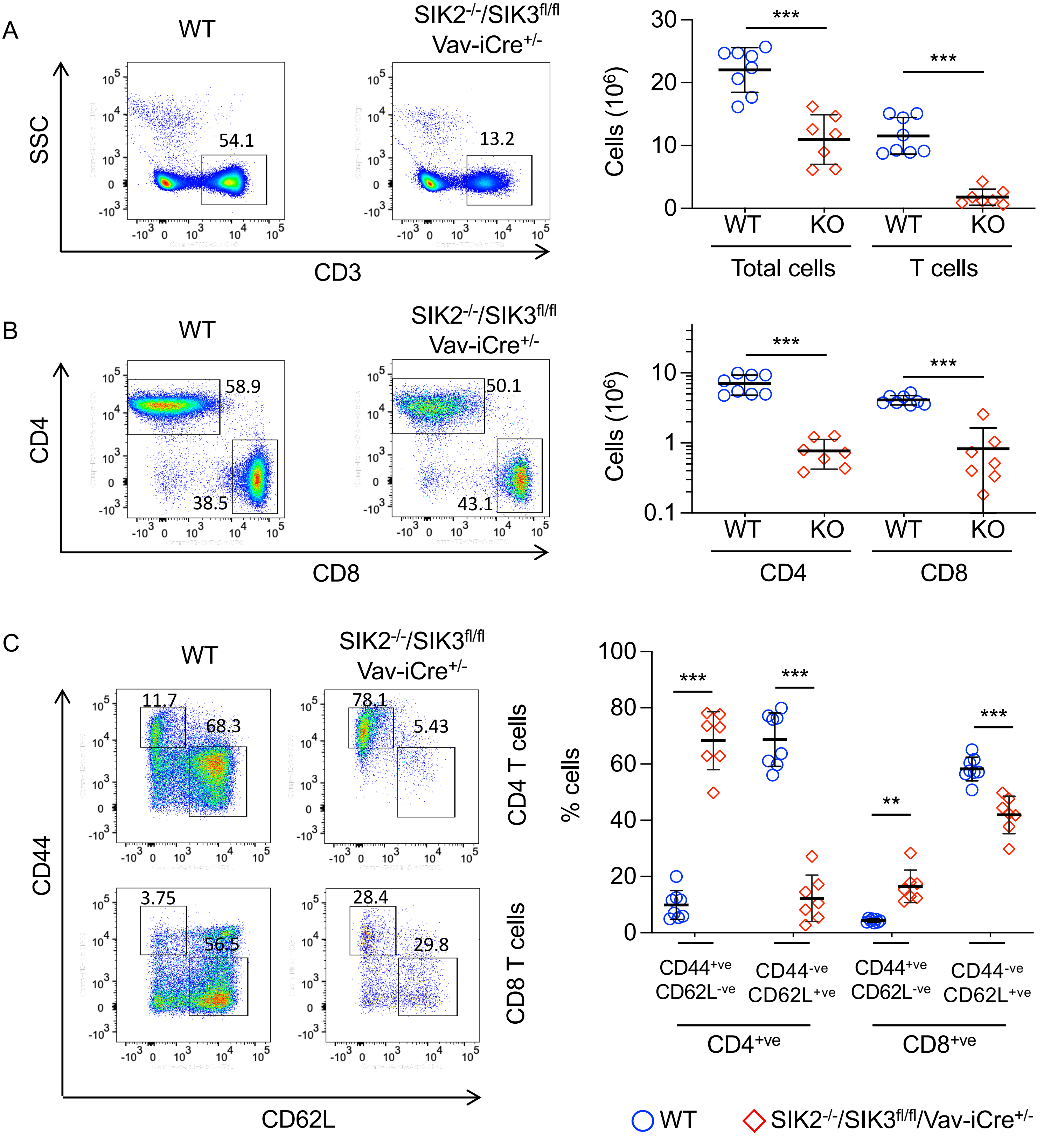

Supplemental Figure 4. Analysis of T cells in SIK2$^{-/-}$/SIK3$^{fl/fl}$/Vav-iCre$^{+/-}$ lymph nodes.
A-C) Lymph nodes (LN) were isolated from wild type (WT, n=8) and SIK2$^{-/-}$/SIK3$^{fl/fl}$/Vav-iCre$^{+/-}$ (KO, n=7) mice and analysed by flow cytometry. Single cell suspensions were analysed for expression of CD3, CD4, CD8, CD44 and CD62L. Total cell counts and numbers of CD3^{+ve} T cells in the LN, along with representative FACS plots are shown in (A). Absolute numbers of CD4 and CD8 T cells along with representative CD4 / CD8 plots of CD3^{+ve} T cells are shown in (B). The expression of CD44 and CD62L was also examined in both CD3^{+ve}/CD4^{+ve} and CD3^{+ve}/CD8^{+ve} T cells and data shows representative flow cytometry plots and the percentage of CD44^{+ve}/CD62L^{-ve} and CD44^{-ve}/CD62L^{+ve} cells (C). Graphs show mean and standard deviation with individual mice indicated by symbols. Differences between wild type and knockout mice were analysed by Student's t-test (A) or RM two way ANOVA and Sidak's post hoc testing (B and C). $p < 0.01$ is indicated by ** and < 0.001 by ***.

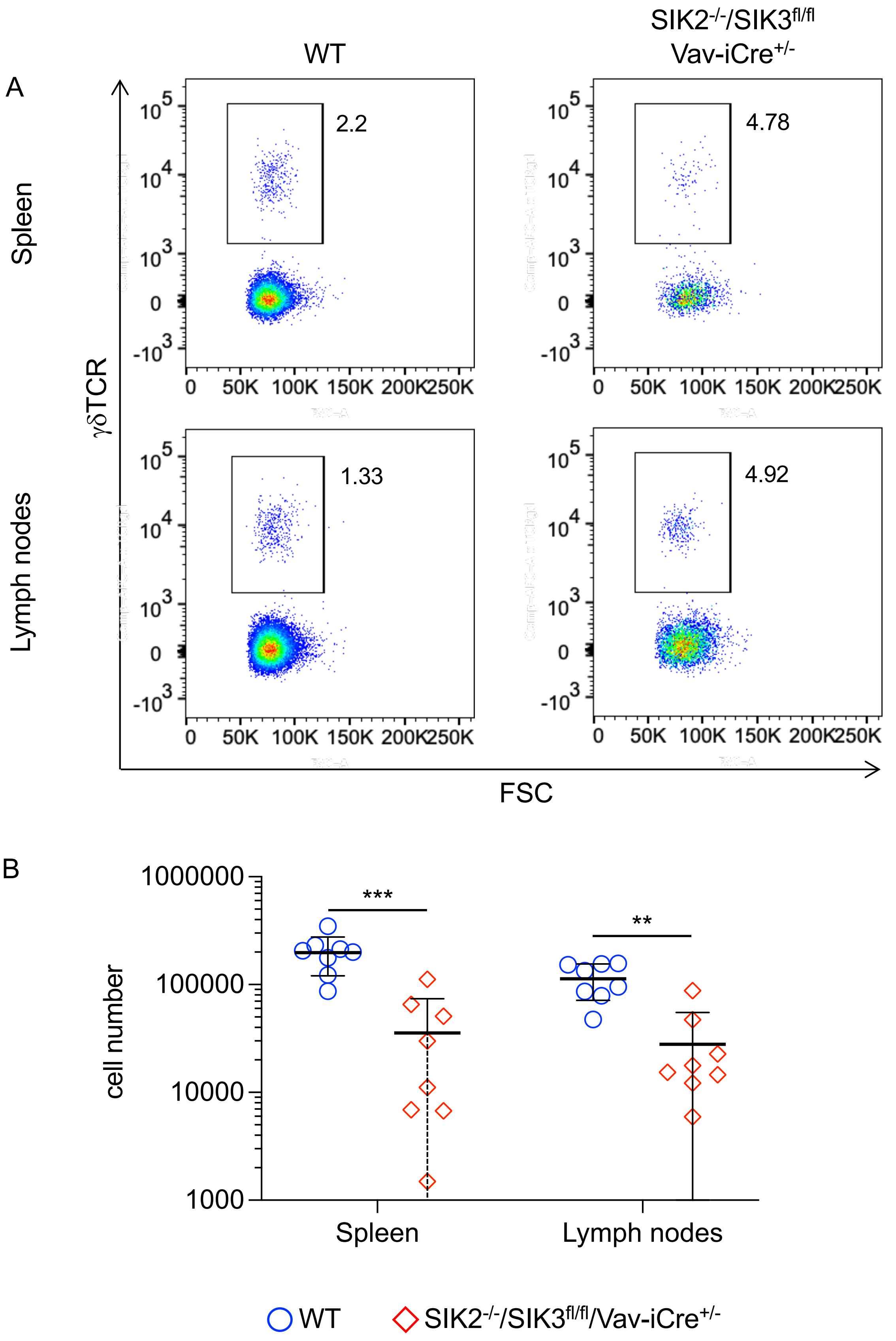

Supplemental Figure 5. γδT cell numbers in wild type and SIK2$^{-/-}$/SIK3$^{fl/fl}$/Vav-iCre$^{+/-}$ mice.
Spleen and lymph nodes were isolated from wild type (n=8) and SIK2$^{-/-}$/SIK3$^{fl/fl}$/Vav-iCre$^{+/-}$ (n=8) mice and analysed by flow cytometry. T cells were identified as CD3^{+ve} cells and γδT cells identified by gating for cells positive for cell surface γδTCR in the CD3^{+ve} gate. Representative plots are shown in (A) and quantification of γδT cell numbers in (B). Data shows mean and standard deviation with individual mice indicated by symbols. Differences between wild type and knockout mice were analysed by RM two way ANOVA followed by Sidak's post hoc testing. $p < 0.01$ is indicated by ** and $p < 0.001$ by ***.

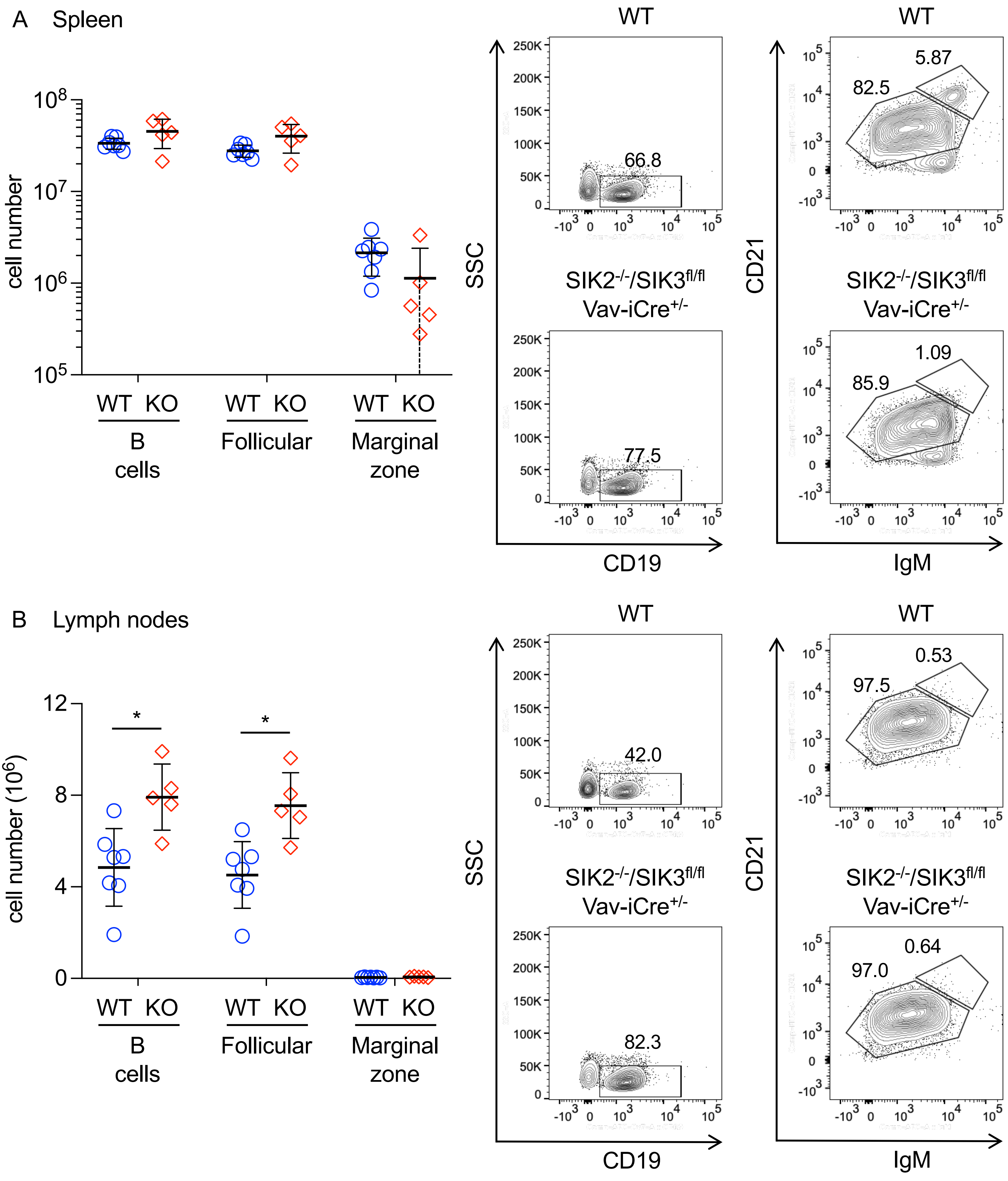

Supplemental Figure 6. B cell numbers in wild type and SIK2$^{-/-}$/SIK3$^{fl/fl}$/Vav-iCre$^{+/-}$ mice. Spleen (A) and lymph nodes (B) were isolated from wild type (WT, n=7) and SIK2$^{-/-}$/SIK3$^{fl/fl}$/Vav-iCre$^{+/-}$ (KO, n=5) mice and analysed by flow cytometry. B cells were identified as CD19^{+ve} cells. B cells were further divided into follicular (IgM^{int}/CD21^{int}) and marginal zone (IgM^{hi}/CD21^{hi}) subsets. Representative flow cytometry plots are shown in the right hand panels and absolute cell numbers in the graphs. Data shows mean and standard deviation with individual mice indicated by symbols. Differences between wild type and knockout mice were analysed by RM two way ANOVA followed by Sidak's post hoc testing. $p < 0.05$ is indicated by *.

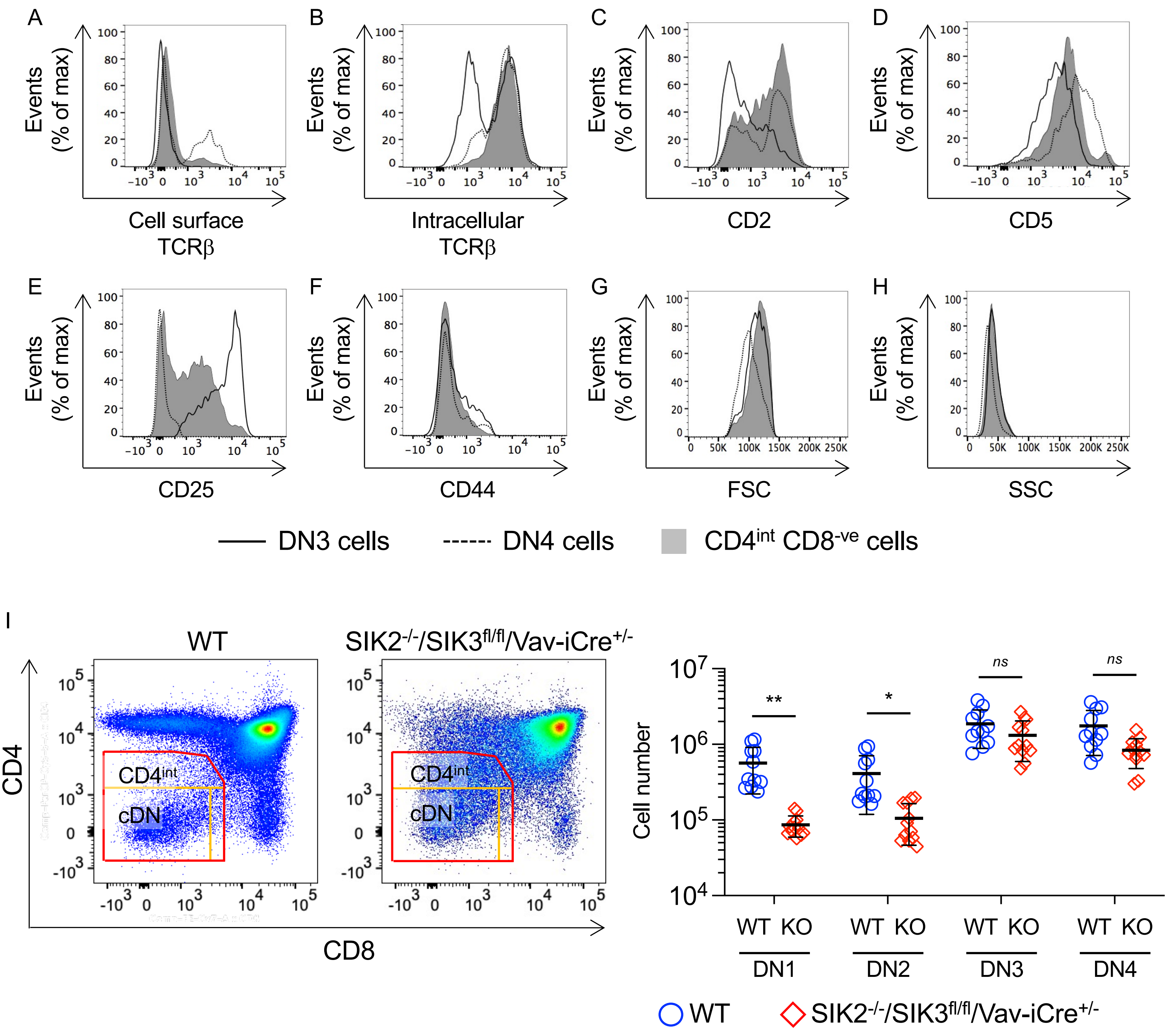

Supplemental Figure 7. Characterisation of $CD4^{int}CD8^{-ve}$ cells in $SIK2^{-/-}/SIK3^{fl/fl}/Vav\text{-}iCre^{+/-}$ thymi.
A-H) Thymi were isolated from wild type (WT, n=11) and $SIK2^{-/-}/SIK3^{fl/fl}/Vav\text{-}iCre^{+/-}$ (KO, n=12) mice and analysed by flow cytometry. Cells were gated for DN3 ($Thy1.2^{+ve}$ $CD4^{-ve}$ $CD8^{-ve}$ $CD44^{-ve}$ $CD25^{+ve}$) and DN4 ($Thy1.2^{+ve}$ $CD4^{-ve}$ $CD8^{-ve}$ $CD44^{-ve}$ $CD25^{-ve}$) populations as well as a $Thy1.2^{+ve}$ $CD4^{int}$ $CD8^{-ve}$ population (see Fig 5A). Histograms show the staining for cell surface and intracellular levels of TCRβ (A,B), cell surface CD2 (C), CD5 (D), CD25 (E) and CD44 (F) as well as forward and side scatter (G, H).
I) The DN gate was expanded to include the $CD4^{int}/CD8^{-ve}$ cells observed in the $SIK2^{-/-}/SIK3^{fl/fl}/Vav\text{-}iCre^{+/-}$ mice (red gate on flow cytometry plots). The original DN (cDN) and $CD4^{int}/CD8^{-ve}$ gate are indicated for comparison. Absolute numbers of $CD44^{+ve}/CD25^{-ve}$ (DN1), $CD44^{+ve}/CD25^{+ve}$ (DN2), $CD44^{-ve}/CD25^{+ve}$ (DN3) and $CD44^{-ve}/CD25^{-ve}$ (DN4) are shown in the graph in the right hand panel. The graph shows mean and standard deviation with individual mice indicated by symbols. Differences between wild type and knockout mice were analysed by two way ANOVA. $p < 0.05$ is indicated by * and < 0.01 by **, ns indicates $p > 0.05$.

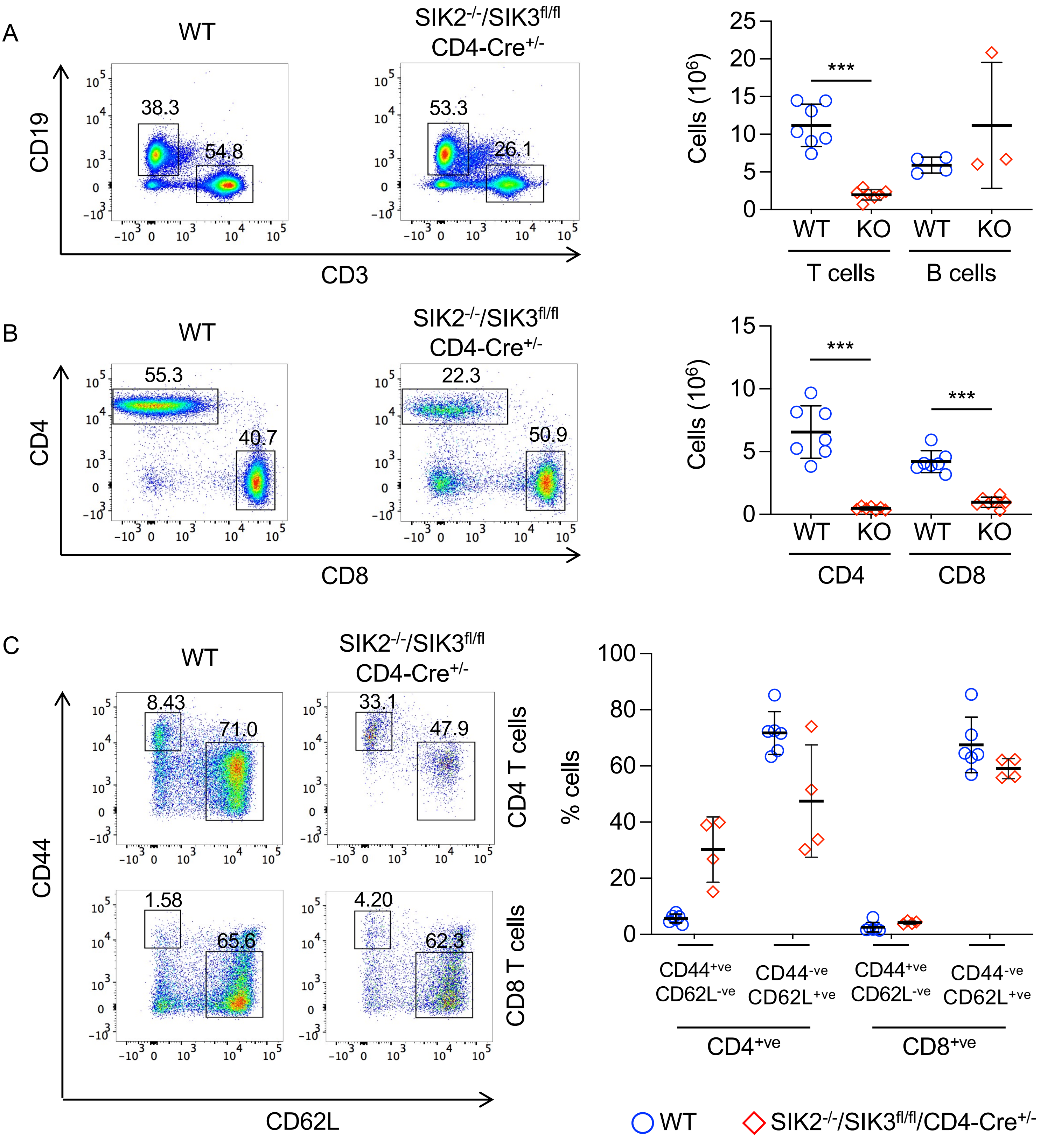

Supplemental Figure 8. Analysis of T cells in SIK2$^{-/-}$/SIK3$^{fl/fl}$/CD4-Cre$^{+/-}$ lymph nodes.
A-C) Lymph nodes (LN) were isolated from wild type (WT) and SIK2$^{-/-}$/SIK3$^{fl/fl}$/CD4-Cre$^{+/-}$ (KO) mice and analysed by flow cytometry for expression of CD3, CD4, CD8, CD44 and CD62L. In a subset of mice, B cells were additionally identified by staining for CD19. Numbers of CD3^{+ve} T cells and CD19^{+ve} B cells in the LN, along with representative FACS plots are shown in (A). Absolute numbers of CD4 and CD8 T cells along with representative CD4 / CD8 plots of CD3^{+ve} T cells are shown in (B). Graphs in (A-B) show mean with symbols representing measurements from individual mice. (C) The expression of CD44 and CD62L was also examined in both CD3^{+ve}/CD4^{+ve} and CD3^{+ve}/CD8^{+ve} T cells and data shows representative flow cytometry plots of the percentage of CD44^{+ve}/CD62L^{-ve} and CD44^{-ve}/CD62L^{+ve} cells. Graph shows mean and standard deviation. Differences in cell number between wild type and knockout mice were analysed by Student's t-test (A) or RM two way ANOVA and Sidak's post hoc testing (B and C). $p < 0.001$ between wild type and knockout is indicated by ***. For wild type mice n=7 in A and B and 5 in C, for knockouts n= 5 in A and B and 4 in C.

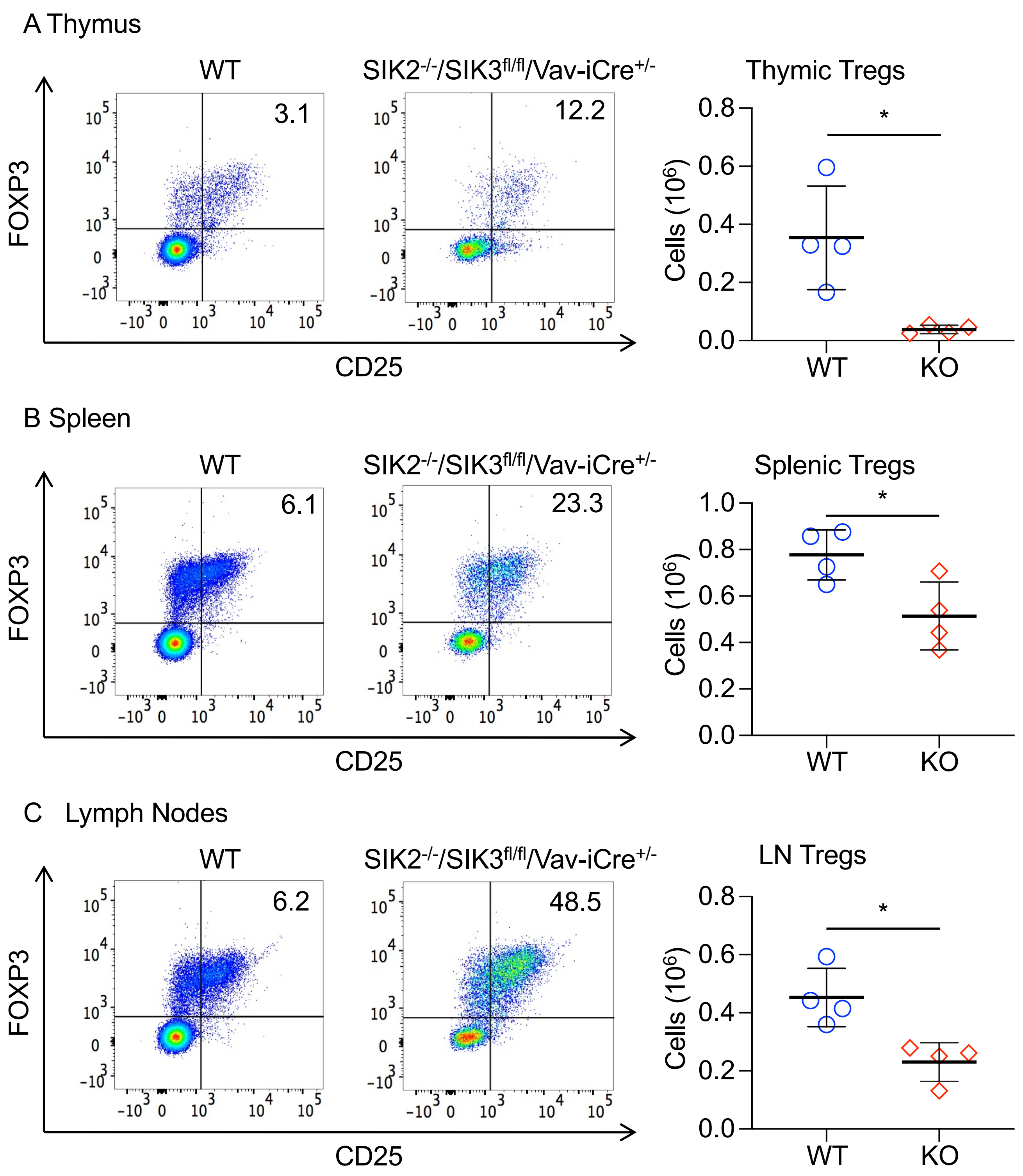

Supplemental Figure 9. Analysis of Tregs in $SIK2^{-/-}/SIK3^{fl/fl}/Vav\text{-}iCre^{+/-}$ mice.
Thymi, spleen and lymph nodes were isolated from wild type (WT, n=4) and $SIK2^{-/-}/SIK3^{fl/fl}/Vav\text{-}iCre^{+/-}$ (KO, n=4) mice. Cells were stained for TCRβ, CD4, CD25 and FoxP3. Tregs were identified as $TCR\beta^{+ve}/CD4^{+ve}/CD25^{+ve}/FoxP3^{+ve}$ cells. Representative CD25 / FoxP3 plots of live gated $TCR\beta^{+ve}/CD4^{+ve}$ cells along with the absolute numbers of Tregs are shown for the thymus (A), spleen (B) and lymph nodes (C). Differences in cell number between wild type and knock-out mice were analysed by two tailed Student's t-test. $p < 0.05$ is indicated by *.

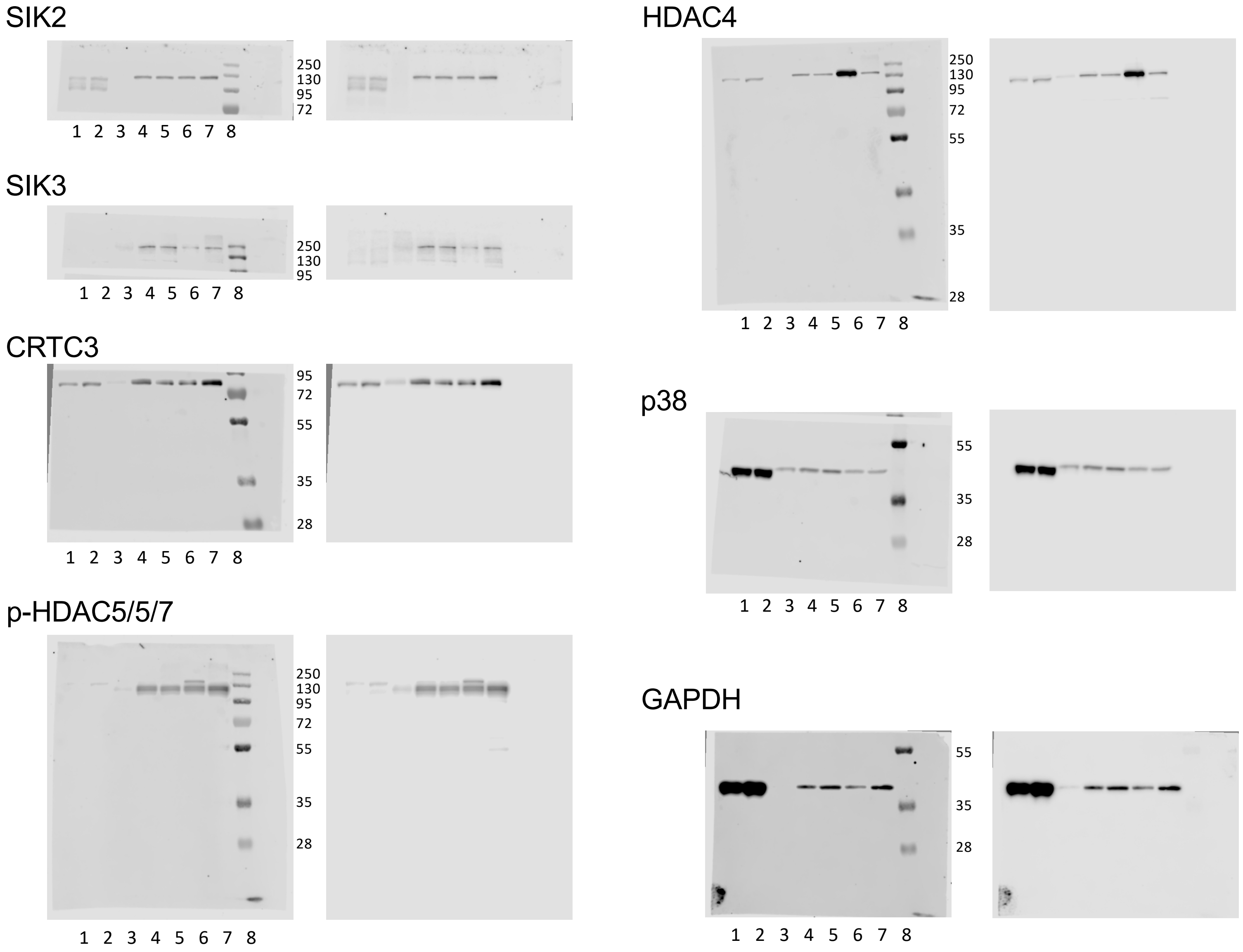

Lane	lysate
1	Bone marrow derived macrophages
2	Bone marrow derived macrophages
3	SIK2 knockout spleen
4	Thymic CD8 SP cells
5	Thymic CD4 SP cells
6	Thymic DN cells
7	Thymic DP cells
8	Molecular weight markers

Supplemental data. Images of blots used for Figure 1

Full length scans of the blots used in figure 1 are shown. Blots were imaged using a Li-Cor Odyssey Fc imaging system and visualised in Image studio. Scans were obtained for both the chemiluminescence channel (to image the antibody signal) and fluorescent channels (600 and 700nm to image the pre-stained molecular weight markers); a merged image is shown on the left and the chemiluminescence channel only on the right. For Figure 1, the the image was cropped to lanes 4 to 8. Weights given for the molecular weight markers are in kDa. The images in Figure 1 were from the chemiluminescence only.

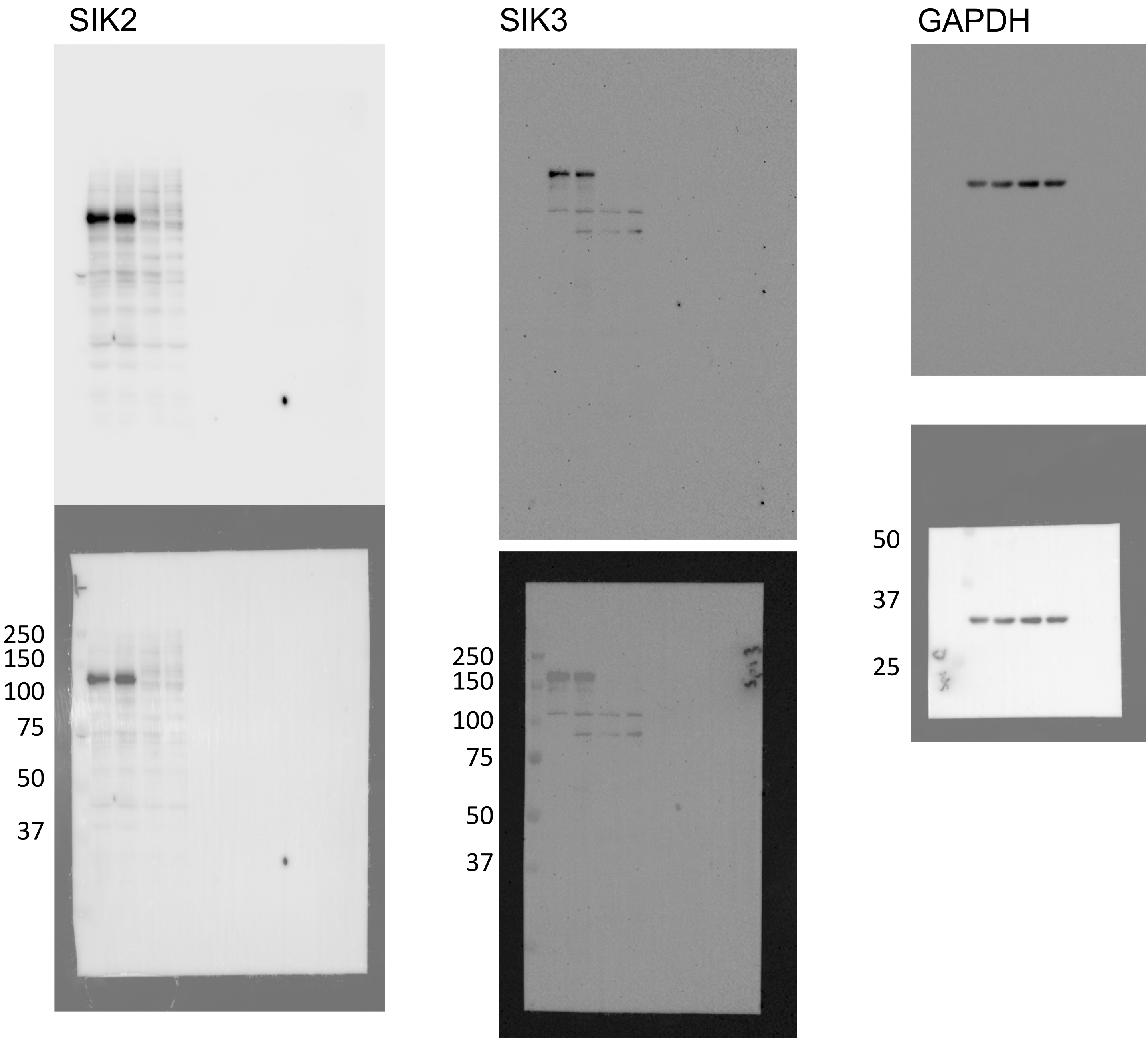

Supplemental data. Images of blots used for supplementary figure 2

Full length scans of the blots used in supplementary figure 2 are shown. Blots were imaged using a Chemidoc MP imaging system from Bio-Rad. Scans were obtained for both the chemiluminescence channel (to image the antibody signal) and colorimetric channel (to image the membrane); the chemiluminescence signal is shown in the top panel and a merged image is shown in the bottom panel. Molecular weights are given in kDa. For supplementary figure 2 images were prepared from the chemiluminescence channel only.
